# miR299a-5p promotes renal fibrosis by suppressing the antifibrotic actions of follistatin

**DOI:** 10.1038/s41598-020-80199-z

**Published:** 2021-01-08

**Authors:** Neel Mehta, Renzhong Li, Dan Zhang, Asfia Soomro, Juehua He, Ivan Zhang, Melissa MacDonald, Bo Gao, Joan C. Krepinsky

**Affiliations:** 1 Division of Nephrology, Department of Medicine, McMaster University, Hamilton, Canada; 2 St. Joseph’s Hospital, 50 Charlton Ave East, Rm T3311, Hamilton, ON L8N 4A6 Canada

**Keywords:** Chronic kidney disease, Renal fibrosis, Glomerular diseases, miRNAs, Locked nucleic acid

## Abstract

Caveolin-1 (cav-1), an integral protein of the membrane microdomains caveolae, is required for synthesis of matrix proteins by glomerular mesangial cells (MC). Previously, we demonstrated that the antifibrotic protein follistatin (FST) is transcriptionally upregulated in cav-1 knockout MC and that its administration is protective against renal fibrosis. Here, we screened cav-1 wild-type and knockout MC for FST-targeting microRNAs in order to identity novel antifibrotic therapeutic targets. We identified that miR299a-5p was significantly suppressed in cav-1 knockout MC, and this was associated with stabilization of the FST 3′UTR. Overexpression and inhibition studies confirmed the role of miR299a-5p in regulating FST expression. Furthermore, the profibrotic cytokine TGFβ1 was found to stimulate the expression of miR299a-5p and, in turn, downregulate FST. Through inhibition of FST, miR299a-5p overexpression augmented, while miR299a-5p inhibition diminished TGFβ1 profibrotic responses, whereas miR299a-5p overexpression re-enabled cav-1 knockout MC to respond to TGFβ1. In vivo, miR299a-5p was upregulated in the kidneys of mice with chronic kidney disease (CKD). miR299a-5p inhibition protected these mice against renal fibrosis and CKD severity. Our data demonstrate that miR299a-5p is an important post-transcriptional regulator of FST, with its upregulation an important pathogenic contributor to renal fibrosis. Thus, miR299a-5p inhibition offers a potential novel therapeutic approach for CKD.

## Introduction

Chronic kidney disease (CKD) is a major cause of morbidity and mortality. It is pathologically characterized by progressive renal fibrosis, which over time results in declining kidney function and ultimately end-stage renal disease^1–3^. Mesangial cells (MC) are specialized pericytes that are involved in the production and secretion of matrix proteins in the glomerulus^4–6^. Activation of MC, with consequent increased production of extracellular matrix proteins contributing to glomerular sclerosis, is well established in CKD and contributes significantly to reduced kidney function^5–8^. Treatments targeted at reversing or slowing matrix production and thus renal fibrosis in CKD are needed.

Caveolae are ubiquitous small (50–100 nm) glycosphingolipid and cholesterol enriched omega-shaped invaginations of the plasma membrane. The caveolin family consists of three proteins, cav-1, cav-2 and cav-3. We have shown that cav-1 is required by MC to produce matrix proteins both basally and in response to profibrotic stimuli such as TGFβ1, high glucose and mechanical stress^9–13^. We further identified significant upregulation of the antifibrotic protein follistatin (FST) in cav-1 deficient MC, and showed it to be important in the reduced glucose and TGFβ-induced profibrotic responses of these cells^14,15^. FST is an ubiquitously expressed secreted glycoprotein that binds to and neutralizes the profibrotic and pro-inflammatory actions of several TGFβ superfamily members, with greatest potency against the activins^16,17^. Therapeutically, our recent studies have shown promising antifibrotic and kidney function preserving effects of recombinant FST in mouse models of diabetic nephropathy and surgically induced CKD^14,15^. While several in vivo and clinical studies have used a single dose of adenoviral (AAV) FST for systemic or local delivery to improve muscle mass, in terms of recombinant protein therapeutics, the short half-life of the FST protein necessitates frequent systemic dosing which is not clinically practical^14,18–20^. Thus, alternative approaches that can more efficiently and stably upregulate FST in vivo may potentially improve the clinical ability to harness the endogenous antifibrotic properties of FST to protect against renal fibrosis.

We have recently shown that increased expression of FST in cav-1 knockout (KO) MC is seen at the transcriptional level^14^. Here, we tested the potential role of microRNA (miRNA)-mediated post-transcriptional regulation of FST. miRNAs, small (~ 22 bp) single-stranded noncoding RNAs, through their complementary “seed sequence” (2–8 bp), bind to a specific miRNA regulatory element (MRE) localized within the 3′ untranslated region (3′ UTR) of the target mRNA^21,22^. Despite multiple targets, mature miRNAs have distinct features that are favorable for their potential therapeutic and biomarker use, including their short sequence and high homology across species^23–26^. Indeed, therapeutically targeting miRNAs using LNA anti-miR technology has become a highly investigated area with both overexpression and inhibition being used depending on the miRNA and its function in disease^23–28^.

In screening for miRNAs targeting FST which are differentially expressed in cav-1 KO and wild-type (WT) MC, we discovered miR299a-5p as an important inhibitor of FST expression. We show that miR299a-5p is profibrotic through its potent ability to repress FST. In a mouse model of CKD, miR299a-5p expression was increased and its inhibition reduced renal fibrosis. Targeting miR299a-5p thus represents a potential therapeutic option for protecting against renal fibrosis in CKD.

## Results

### miR299a-5p expression and activity is downregulated in cav-1 deficient MC

**Figure 1:**
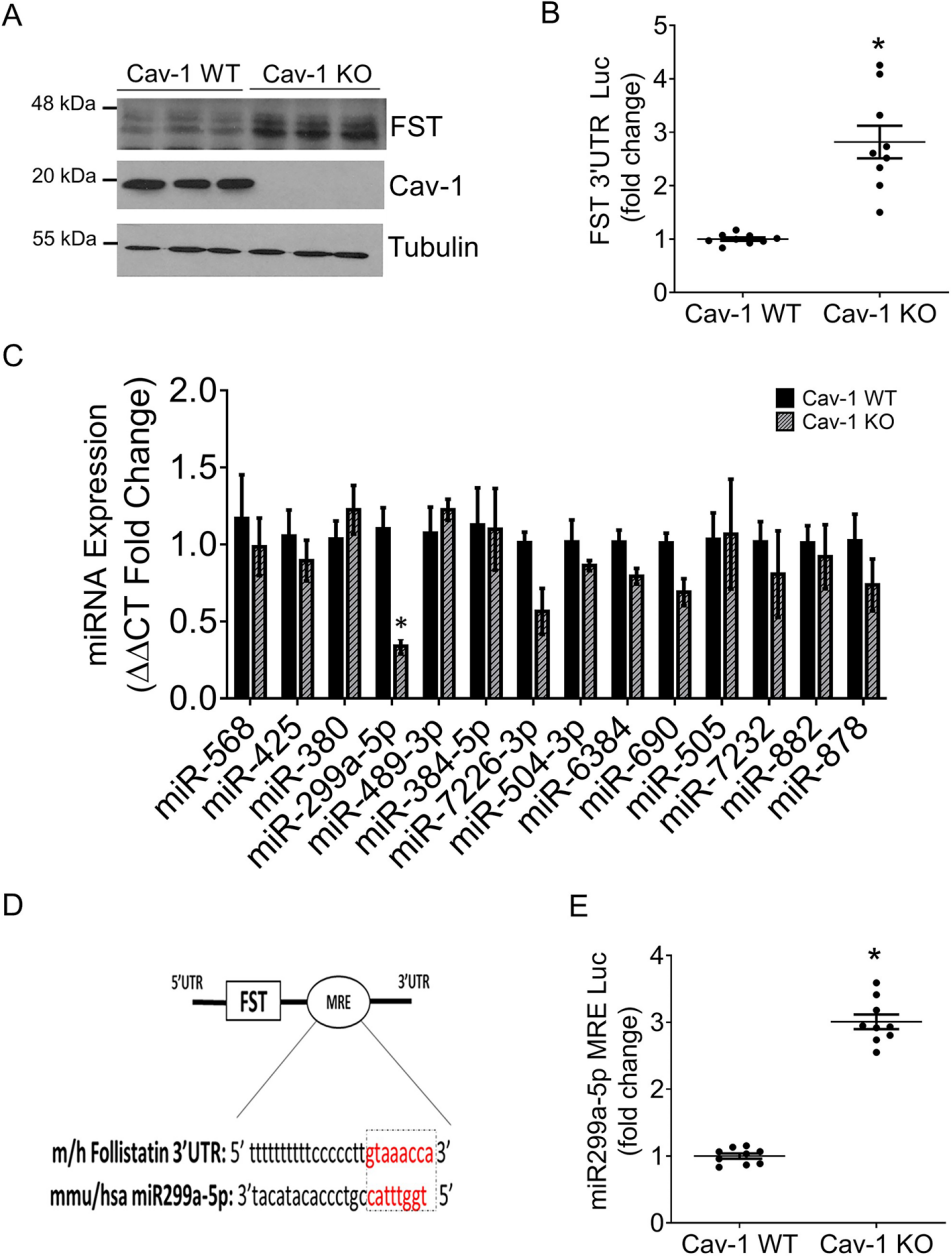
miR299a-5p targets the 3′UTR of FST and its expression is decreased in cav-1 KO MCs. (**A**) FST protein was increased in cav-1 KO MC (n = 9, *p < 0.05). (**B**) Stability of the FST 3′UTR, as assessed by a luciferase reporter construct, was significantly enhanced in cav-1 KO vs WT MC (n = 9, *p < 0.05). (**C**) qRT-PCR analysis of the expression of miRNAs targeting FST showed significantly lower expression of miR299a-5p in cav-1 KO MC (n = 12, *p < 0.05). (**D**) The miR299a-5p 8-mer miRNA regulatory element (MRE) is conserved in the 3′UTR of mouse and human FST. (**E**) A luciferase reporter for miR299a-5p (MRE-luc) showed increased stability, reflecting lower miR299a-5p activity, in cav-1 KO vs WT MC (n = 9, *p < 0.05).

We previously identified FST as the most upregulated gene in cav-1 KO compared to WT MC^14,15^. Here, we first confirmed elevated FST expression at the protein level (Fig. 1A) in cav-1 KO MC, and as shown previously^14^. Confirmation of specificity of the FST antibody (FST-H114) used in these studies for immunoblotting was assessed using FST targeting siRNA (Fig. S1A) and recombinant FST protein (Fig. S1B). Our previous data showed that cav-1 transcriptionally regulates FST through SP1^14^. To determine whether cav-1 is also involved in post-transcriptional regulation of FST, we examined stability of the FST 3′UTR. We observed that cav-1 elimination resulted in significant stabilization of the 3′UTR of FST (Fig. 1B).

The 3′UTR of a gene can be regulated by numerous factors including microRNAs (miRNAs)^29^. miRNAs bind to a complementary target mRNA sequence in the 3′UTR and effectively silence the gene by targeting it for degradation and/or inhibiting its translation into protein^21^. Hypothesizing that cav-1 might destabilize the 3′UTR of FST through altered miRNA expression, we performed a bioinformatics screen to identify potential candidate miRNAs that target the 3′UTR of mouse FST, with candidate miRNAs selected based on the greatest number of hits that were present in several miRNA target prediction algorithms including TargetScan, MicroCosm, mirRDB and miRSearch V3.0. As seen in Fig. 1C, of the 14 miRNAs identified, miR299a-5p was the most reduced in cav-1 KO vs WT MC. Its 8-mer miRNA regulator element (MRE) within the FST 3′UTR is shown in Fig. 1D, with 100% homology between mouse and human in this region. To functionally validate that miR299a-5p is downregulated in cav-1 KO MC, we generated a construct with the miR299a-5p MRE downstream of luciferase. Consequently, stability of the luciferase is indicative of miR299a-5p expression and activity. As expected, given decreased miR299a-5p expression, the miR299a-5p MRE-luc is stabilized in cav-1 KO compared to cav-1 WT MC (Fig. 1E). These results suggest that the increased FST 3′UTR stability seen in cav-1 KO MC is mediated by suppressed miR299a-5p expression.

### miR299a-5p attenuates FST expression

**Figure 2:**
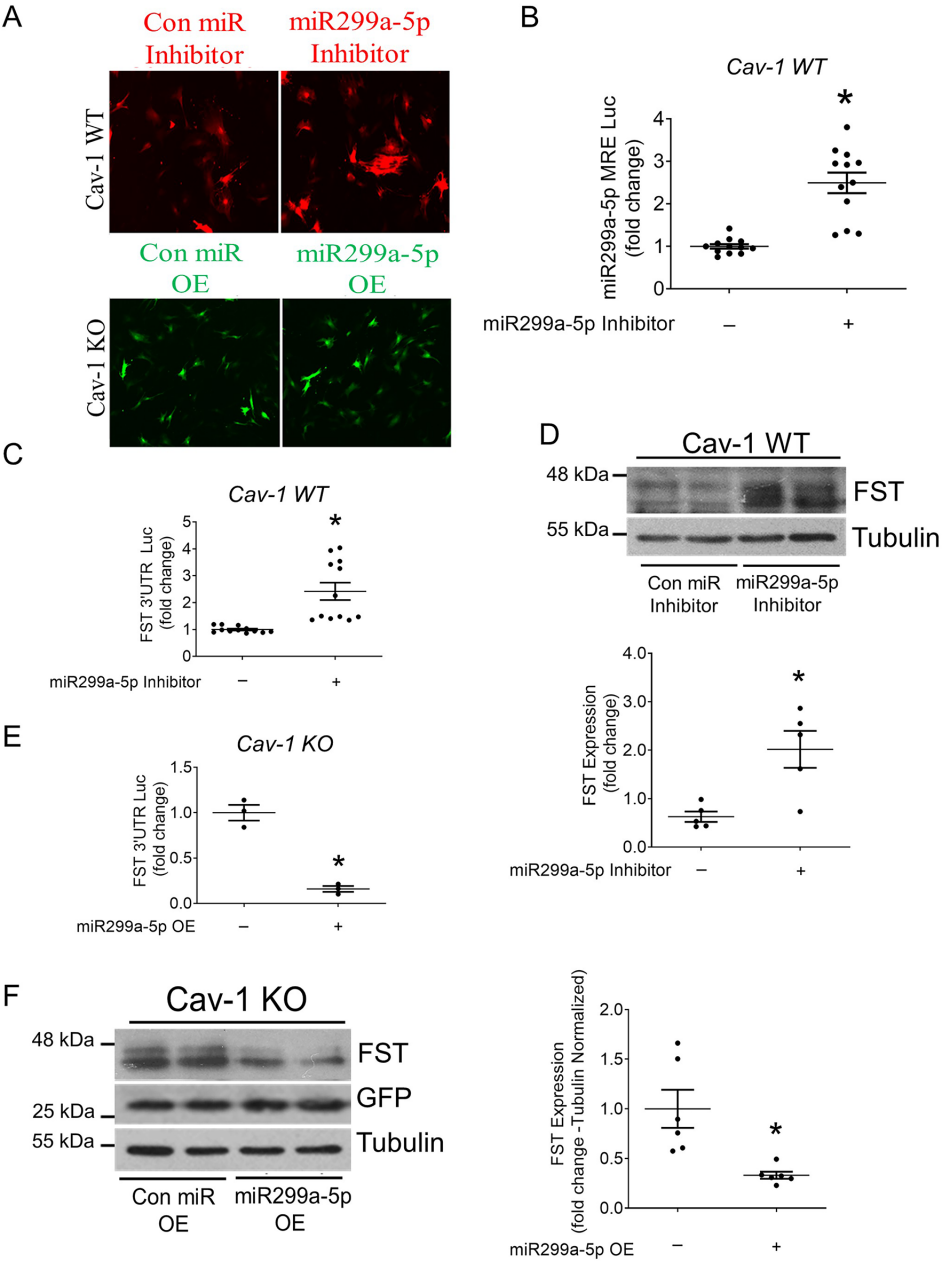
miR299a-5p regulates the expression of FST through its 3′UTR. (**A**) Cav-1 WT and KO MC were transfected with either the miR299a-5p inhibitor or precursor clones, or their respective controls, and effective transfection confirmed with mCherry and GFP immunofluorescence, respectively. (**B**) miR299a-5p inhibition significantly increased stability of the miR299a-5p MRE luciferase reporter in cav-1 WT MC (n = 12, *p < 0.05). (**C**) An miR299a-5p inhibitor significantly enhanced stability of the FST 3′UTR in cav-1 WT MC (n = 12, *p < 0.05). (**D**) miR299a-5p inhibition significantly increased FST protein expression in cav-1 WT MC (n = 5, *p < 0.05). (**E**) Overexpression (OE) of a miR299a-5p precursor significantly repressed stability of the FST 3′UTR in KO MC. (n = 3, *vs control, p < 0.05). (**F**) miR299a-5p OE in cav-1 KO MC significantly decreased FST protein expression (n = 6, *p < 0.05, normalized to tubulin).

Based on the observation that WT cells express significantly higher levels and activity of miR299a-5p compared to KO cells, to directly validate the role of miR299a-5p in destabilizing the FST 3′UTR in WT MC and to confirm whether miR299a-5p is sufficient in destabilizing the FST 3′UTR, we tested the effects of miR299a-5p inhibition in WT cells and miR299a-5p overexpression in KO cells. Inhibition in WT cells was achieved using a miR299a-5p inhibitor construct which, upon transfection and post-transcriptional processing, results in the formation of an entrapping structure that attracts and binds to two miR299a-5p molecules, thus preventing miR299a-5p activity via inhibiting binding to target mRNAs, without reducing miR expression. A miR299a-5p precursor construct was used for miR299a-5p overexpression in KO cells. Briefly, upon transfection, precursor miR299a-5p is matured using the endogenous cellular miRNA processing machinery. Additionally, the inhibitor or precursor constructs also produce mCherry or GFP proteins, respectively, allowing assessment of transfection by immunofluorescence (Fig. 2A). Functionality of the miR299a-5p inhibitor was confirmed by observing significantly increased miR299a-5p MRE-luc stability in WT cells (Fig. 2B); miR299a-5p overexpression was confirmed by PCR in KO cells (Sup Fig. S2A).

As hypothesized, miR299a-5p inhibition significantly increased stability of the FST 3′UTR in cav-1 WT MC (Fig. 2C) and in turn also increased the protein expression of FST (Fig. 2D). Conversely, miR299a-5p overexpression significantly destabilized the FST 3′UTR in cav-1 KO (Fig. 2E). Overexpression of miR299a-5p also significantly reduced FST protein expression in cav-1 KO MC (Fig. 2F), with similar effects observed in cav-1 WT MC (data not shown). These results clearly show a direct role for miR299a-5p in post-transcriptionally regulating the expression of FST.

### TGFβ1-dependent repression of FST is mediated by miR299a-5p

**Figure 3:**
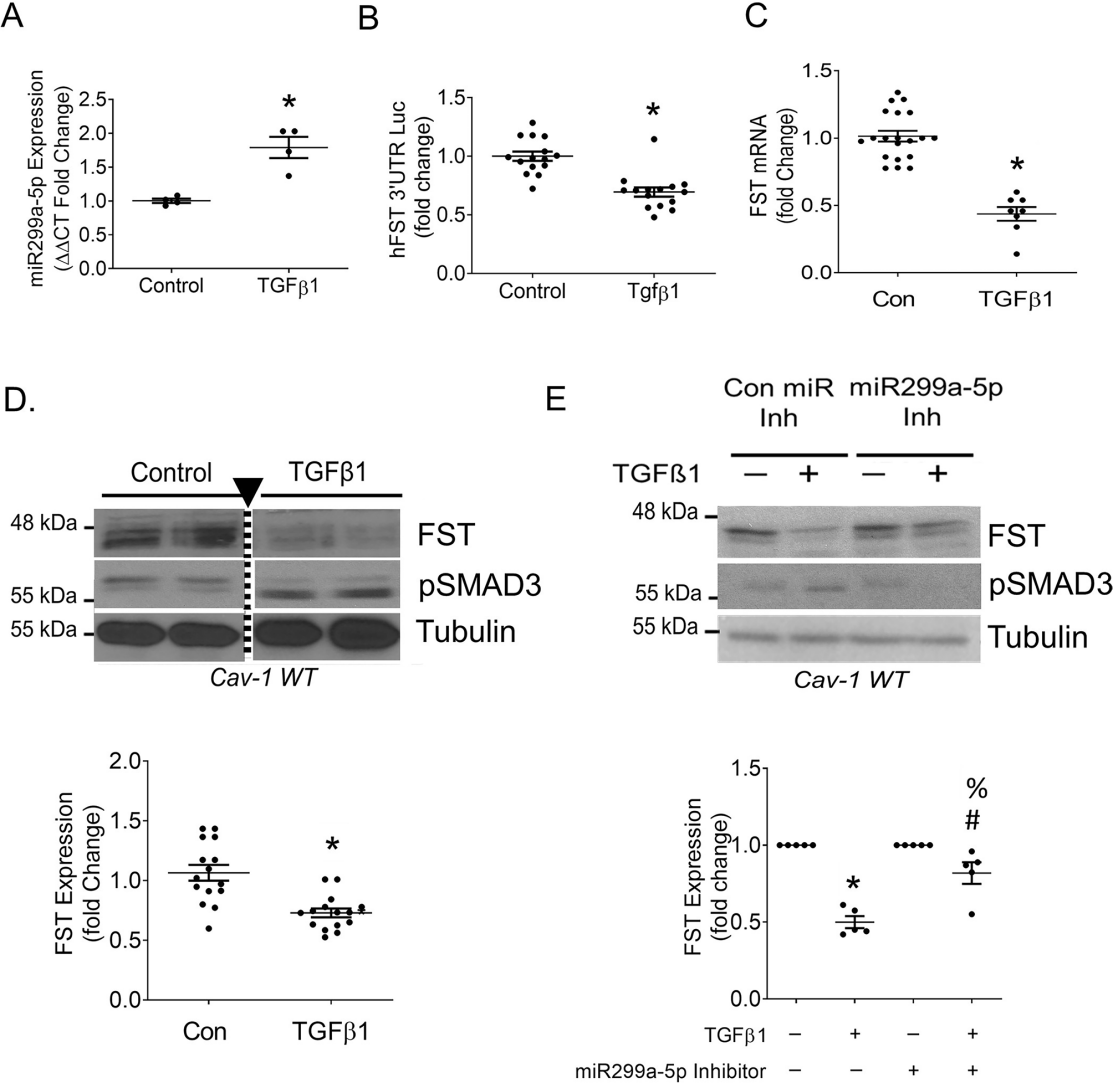
TFGβ1 represses FST through miR299a-5p upregulation in cav-1 WT MC. (**A**) TGFβ1 (5 ng, 24 h) increased the expression of miR299a-5p (n = 4, *p < 0.05) and (**B**) decreased stability of the FST 3′UTR (n = 15, *p < 0.05). (**C**) TGFβ1 (5 ng, 24 h) reduced FST mRNA transcript (n = 8, *p < 0.05) (**D**) TGFβ1 (5 ng, 24 h) reduced FST protein expression (n = 4, *p < 0.05), while inducing phosphorylation of Smad3, triangle pointing to the dotted line indicates an edit made on the immunoblot image, where additional lanes not relevant to the experiment were cropped out, full gel provided in Sup Fig. S8). (**E**) Inhibition of miR299a-5p blunted the ability of TGFβ1 (5 ng, 24 h) to reduce FST protein expression, while reducing the phosphorylation of Smad3 (n = 5, *vs con inh-con, #vs miR inh-con, %vs con inh-TGFβ1, p < 0.05).

TGFβ1 is a major profibrotic factor in the development and progression of CKD^30–33^. FST binds to and neutralizes the actions of several TGFβ family members, primarily activins. While not directly capable of binding and neutralizing TGFβ1, FST was shown to inhibit the TGFβ1-induced profibrotic response in numerous cell types, including MC^14^. Smad3 is the major signaling mediator of both TGFβ1 and activins. Interestingly, recent evidence has shown Smad3-dependent induction of miR-154a, a miR299a-5p family member^34–36^ We have not seen any difference in the expression of TGFβ1 between WT and KO cells (data not shown). However, whether TGFβ1 signaling regulates miR299a-5p expression in MC is not known. We thus questioned whether TGFβ1 also increases the expression of miR299a-5p in MC^34–36^. Indeed, we observed that TGFβ1 prominently increased the miR299a-5p transcript in WT MC (Fig. 3A). This was associated with significant destabilization of the FST 3′UTR (Fig. 3B) as well as decreased FST transcript (Fig. 3C) and protein expression (Fig. 3D). We next determined whether miR299a-5p induction mediated the FST downregulation by TGFβ1. As predicted, inhibition of miR299a-5p activity attenuated the downregulation of FST by TGFβ1 (Fig. 3E). To assess for functionality, we determined whether secretion of the primary pro-fibrotic cytokine neutralized by FST, Activin A, is induced by TGFβ1 and affected by miR299a-5p. Interestingly, we found that inhibition of miR299a-5p in WT MC prevents TGFβ1-induced Activin A secretion (Sup Fig. S2B), while miR299a-5p overexpression in KO MC augments TGFβ1-induced Activin A secretion (Sup Fig. S2C). These results collectively illustrate that TGFβ1-induced miR299a-5p expression leads to FST downregulation, with consequent effects on Activin A availability.

### miR299a-5p augments the profibrotic effects of TGFβ1 through FST downregulation

**Figure 4:**
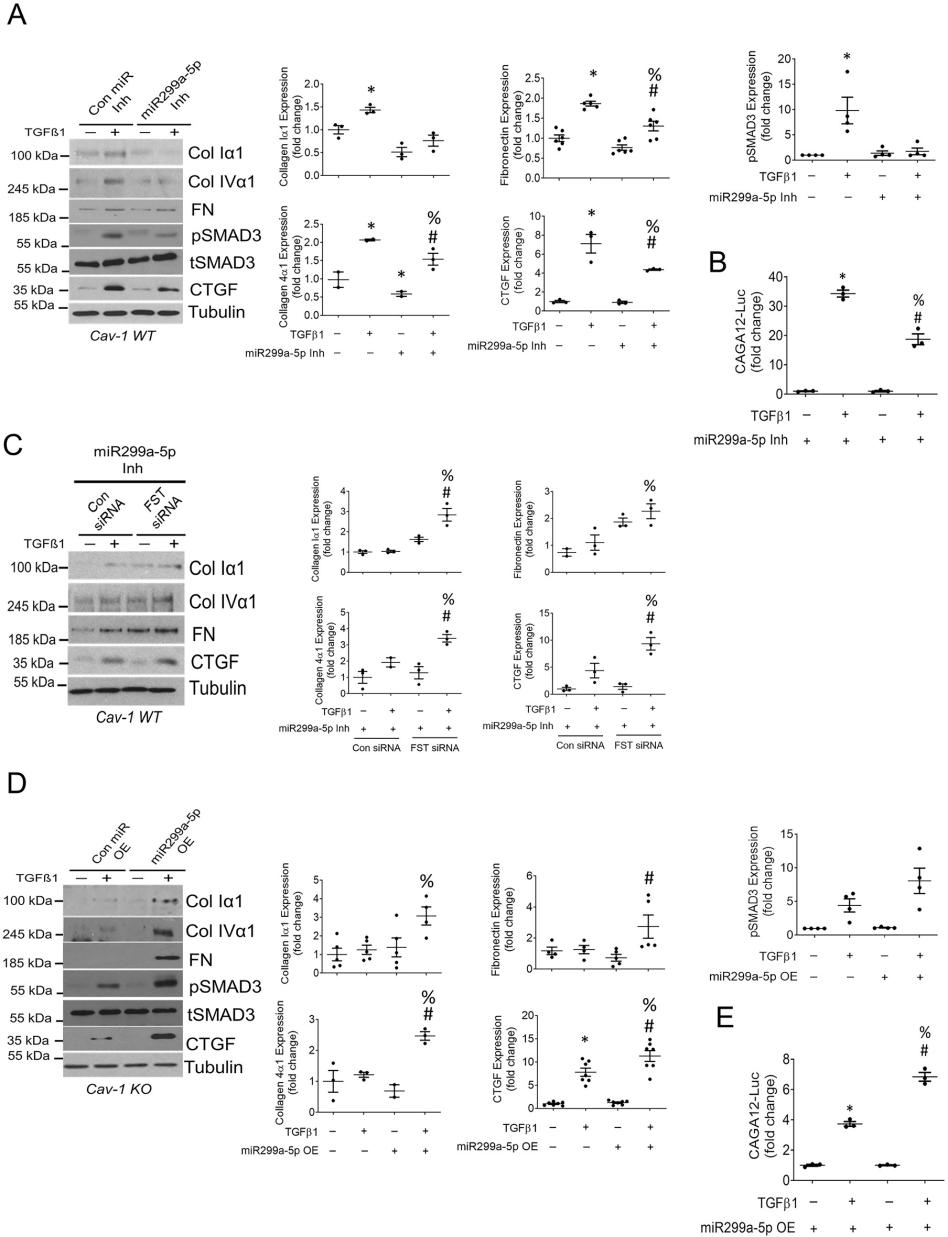
TGFβ1 upregulation of miR299a-5p enables ECM production through FST downregulation. (**A**) miR299a-5p inhibition blunted basal ECM and CTGF expression and decreased TGFβ1 (0.5 ng, 24 h)-induced ECM and CTGF production as well as Smad3 phosphorylation in cav-1 WT MC (n = 3, *vs con inh-con, #vs miR inh-con, %vs con inh-TGFβ1, p < 0.05). (**B**) miR299a-5p inhibition blunted TGFβ1-induced Smad3 signaling, assessed through activation of the Smad3-responsive CAGA_12_ luciferase (n = 3, *vs con inh-con, #vs miR inh-con, %vs con inh-TGFβ1, p < 0.05). (**C**) FST downregulation with siRNA attenuated the ability of an miR-299a-5p inhibitor to reduce the profibrotic response to TGFβ1 (0.5 ng, 24 h) (n = 3, *vs con siRNA-con, #vs FST siRNA-con, %vs con siRNA-TGFβ1, p < 0.05). (**D**) Overexpression (OE) of miR299a-5p significantly enhanced TGFβ1 (0.5 ng, 24 h)-induced production of ECM and CTGF production while augmenting the phosphorylation of Smad3 in cav-1 KO MC (n = 3, *vs con OE-con, #vs miR OE-con, %vs con OE-TGFβ1, p < 0.05). (**E**) miR299a-5p overexpression augmented TGFβ1-induced Smad3 signaling, assessed through activation of the Smad3-responsive CAGA_12_ luciferase (n = 3, *vs con OE-con, #vs miR OE-con, %vs con OE-TGFβ1, p < 0.05).

Given our findings that TGFβ1 reduces the expression of FST through miR299a-5p upregulation, and the known ability of FST to inhibit TGFβ1 profibrotic effects^17,37–39^, we hypothesized that miR299a-5p contributes to the TGFβ1 profibrotic response in MC via the profibrotic Smad3 signaling pathway. We first inhibited miR299a-5p expression in cav-1 WT MC, and observed that this significantly blunted the ability of TGFβ1 to induce extracellular matrix (ECM) proteins and the profibrotic cytokine connective tissue growth factor (CTGF) (Fig. 4A). To determine whether this protective effect was due to increased FST expression, we inhibited both miR299a-5p and FST, the latter using siRNA. As expected, the downregulation of FST using FST-targeting siRNA (Sup Fig. S1A), in the presence of miR299a-5p inhibition, enabled TGFβ1 induction of ECM protein and CTGF production (Fig. 4C). miR299a-5p inhibition also blunted TGFβ1-induced Smad3 activation, as seen through pSmad3 immunoblotting (Fig. 4A) and assessment of Smad3 transcriptional activity using the Smad3 reporter CAGA-12 luciferase (Fig. 4B). These results show that miR299a-5p mediates TGFβ1 profibrotic responses through repression of FST and consequent effects on Smad3 activation.

We previously showed that cav-1 KO MC exhibit significantly decreased ECM protein expression in response to numerous profibrotic stimuli, including TGFβ1^10–13^. We recently showed that the elevated expression of FST by these cells is a key mediator of these findings^15^. Since we now demonstrate that cav-1 KO cells also have significantly decreased miR299a-5p expression and activity (Fig. 1C,E), we assessed whether this contributes to their antifibrotic phenotype. We thus overexpressed miR299a-5p in cav-1 KO MC, and showed that this augmented their activation of Smad3 and profibrotic responses to TGFβ1 (Fig. 4D,E), with similar augmentation of TGFβ1-induced profibrotic signals also observed in cav-1 WT MC (data not shown).

### miR299a-5p expression is increased in mice with CKD, and its inhibition protects against renal fibrosis

**Figure 5:**
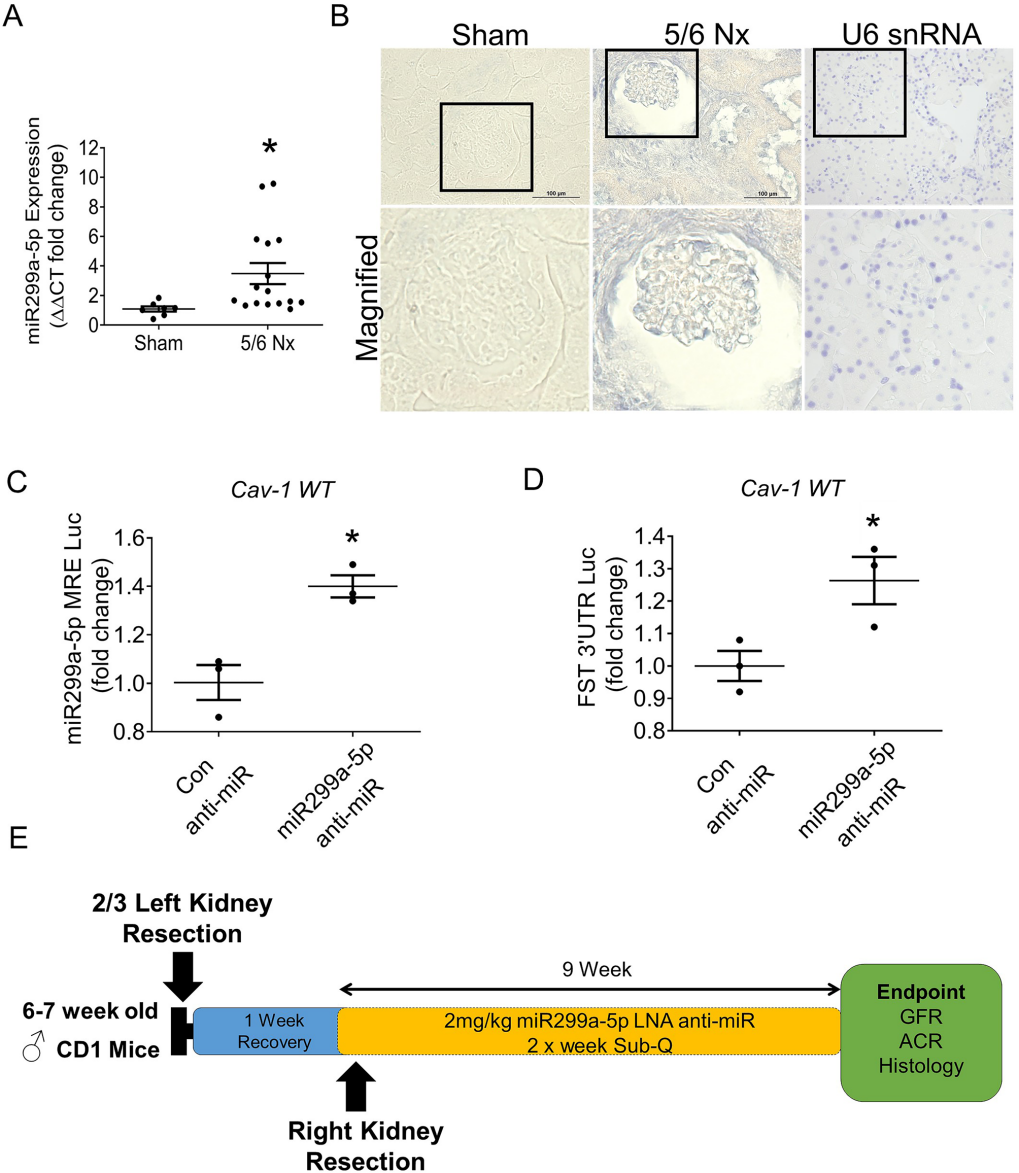
miR299a-5p expression is elevated in the kidneys of mice with CKD. The expression of miR299a-5p was strongly elevated in the glomeruli and tubules of mice with the 5/6 nephrectomy model of CKD, as assessed through qRT-PCR (**A**) and in-situ hybridization (ISH), where blue staining represents positive signal. U6 was used as a positive control. Staining in the absence of probe was also carried out as a negative control (images not shown). Magnified regions from the outlined boxes are shown to illustrate glomerular staining (**B**). (**C**) The in vivo miR299a-5p LNA anti-miR inhibitor (100 nM, 24 h) stabilized the MRE-luciferase in cav-1 WT MC (n = 3, *p < 0.05), as well as FST 3′UTR luciferase construct (**D**) (n = 3, *p < 0.05). (**E**) Flowchart of the timeline for in vivo assessment of the anti-fibrotic potential of miR299a-5p inhibition using an in vivo LNA anti-miR inhibitor targeting miR299a-5p in mice with CKD.

TGFβ1 is well established to be increased in the kidneys and serum of mice with CKD^30–33^. Since our in vitro data show that TGFβ1 upregulation of miR299a-5p mediates its profibrotic responses, we examined whether miR299a-5p expression is elevated in the kidneys of mice with CKD. CKD was induced by surgical reduction of 5/6 of the kidney mass. Using both qRT-PCR (Fig. 5A) and miRNA in-situ hybridization (ISH) (Fig. 5B), we show that the expression of miR299a-5p is elevated in glomeruli of mice with CKD. This suggests that miR299a-5p may contribute to fibrosis in CKD.

Next, we sought to determine the therapeutic potential of miR299a-5p inhibition in mice with CKD. Here, we assessed whether inhibition of miR299-5p activity using Locked Nucleic Acid (LNA) anti-miR technology is an effective strategy for protecting against renal fibrosis in vivo using the 5/6 nephrectomy model. The miR299a-5p anti-miR inhibitor does not directly decrease miR levels. Rather, it binds to the miRNA, rendering it unable to bind its mRNA 3′UTR targets. Thus, the efficacy and functionality of the in vivo inhibitor was initially tested in vitro in MC and confirmed to effectively stabilize the miR299a-5p MRE luciferase (Fig. 5C) and FST 3′UTR luciferase (Fig. 5D). After confirming the efficacy of the miR299a-5p anti-miR, mice were then treated with the in vivo grade miRNA LNA anti-miR inhibitor targeting miR299a-5p for 9 weeks after induction of CKD as shown in Fig. 5E.

**Figure 6:**
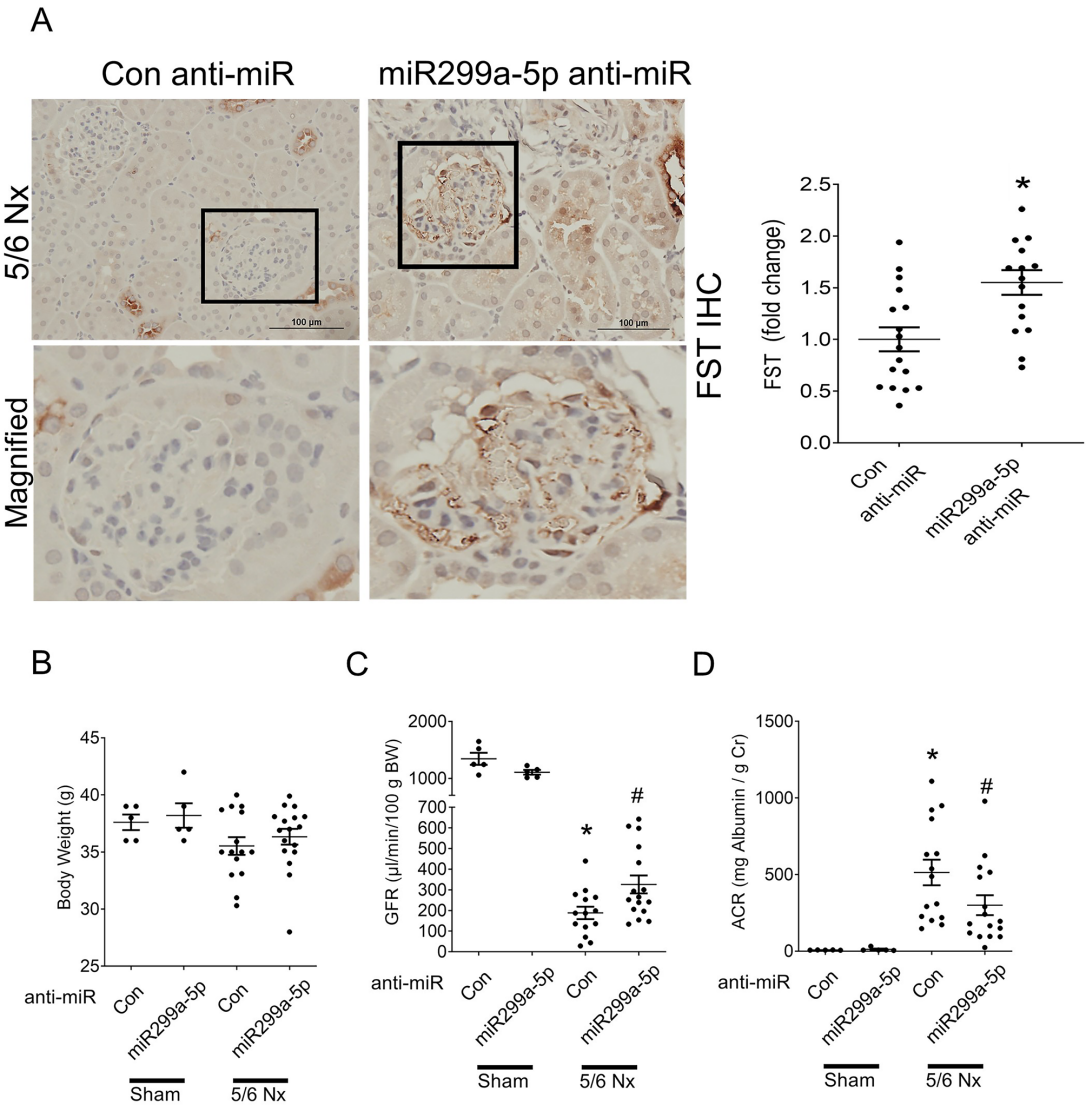
miR299a-5p inhibition increased renal FST expression, improved kidney function and attenuated albuminuria in mice with CKD. (**A**) miR299a-5p anti-miR administration increases the expression of FST in the glomeruli of mice with CKD (*vs 5/6 Nx-Con-anti-miR, p < 0.05, scale bar = 100 μm), magnified regions outlined in black shown below. (**B**) There was no difference in body weight between any of the groups. (**C**) The drastically diminished glomerular filtration rate (GFR) in CKD mice injected with control anti-miR was improved by miR299a-5p LNA administration (*vs Sham-Con-anti-miR, #vs 5/6 Nx-Con-anti-miR, p < 0.05 by t-test). (**D**) Albuminuria, as measured by the urinary albumin to creatinine ratio (ACR), was elevated in CKD mice and improved with miR299a-5p anti-miR administration (*vs Sham-Veh, #vs 5/6 Nx-Con-anti-miR, p < 0.05 by t-test).

**Figure 7:**
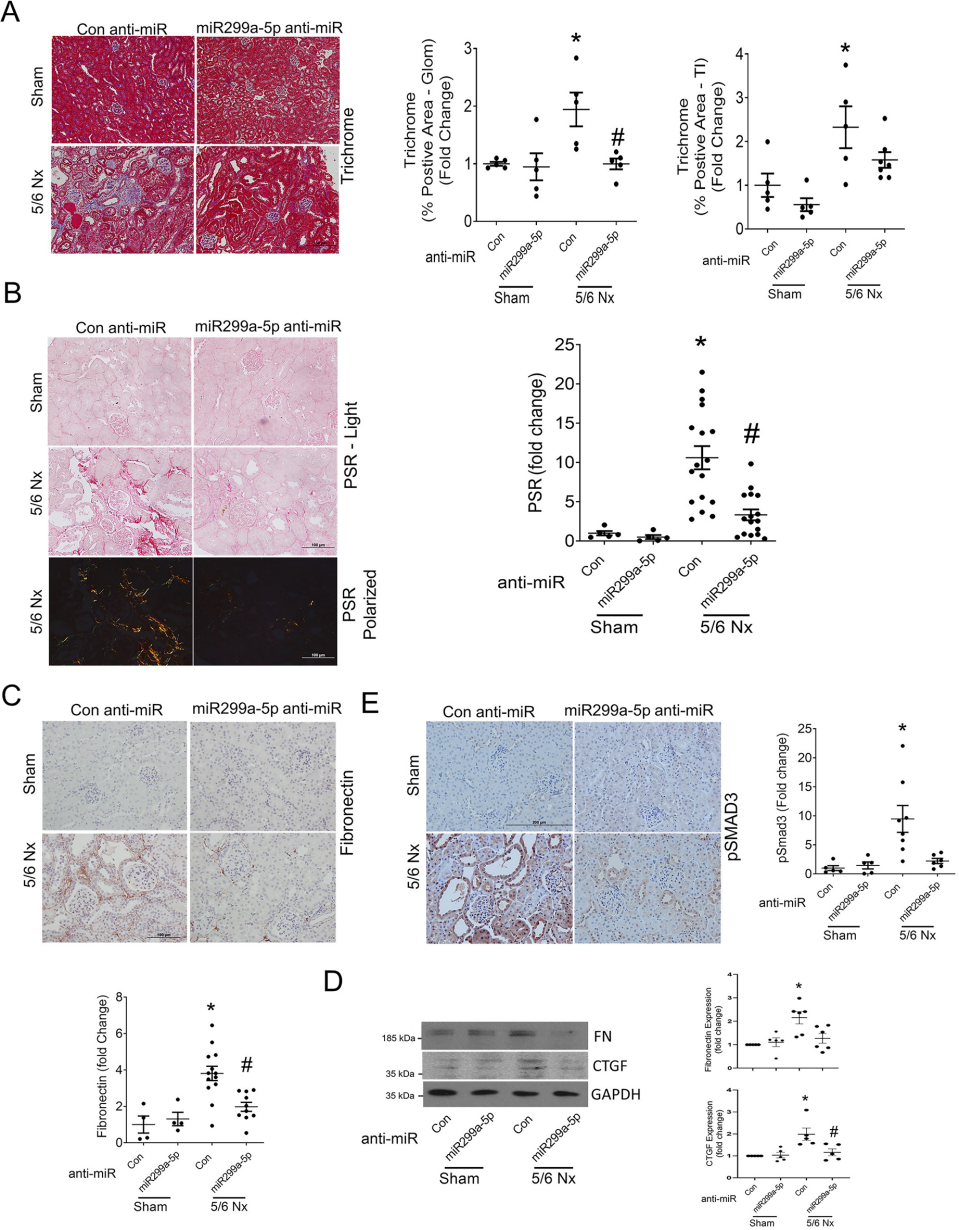
miR299a-5p inhibition reduced renal fibrosis in mice with CKD. CKD mice exhibited extensive glomerular sclerosis and tubulointerstitial fibrosis, as assessed by trichrome (glomerular (glom) and tubular interstitial (TI) areas quantified) (**A**) and PSR (**B**) staining, fibronectin immunohistochemistry (**C**), and fibronectin and CTGF immunoblotting (**D**) all of which were improved by miR299a-5p anti-miR administration. (**E**) Elevated renal Smad3 signaling in mice with CKD, assessed using pSmad3 immunohistochemistry, was improved by miR299a-5p anti-miR administration (*vs Sham-Con-anti-miR, #vs 5/6 Nx-Con anti-miR, p < 0.05 by t-test, scale bar = 100 μm or 200 μm).

First, we assessed renal FST expression to determine whether miR299a-5p inhibition was functional in the kidneys of mice with CKD. As expected, FST expression in the CKD mice treated with miR299a-5p anti-miR was significantly upregulated when compared with control anti-miR treated mice (Fig. 6A). Based on the protective role of FST that we previously observed in this model, we assessed whether miR299a-5p inhibition protects against the progression of CKD^15,40,41^. Firstly, we assessed whether miR299a-5p anti-miR administration led to overt phenotypic changes. Figure 6B shows that at study endpoint, no significant difference in body weight was observed between any of the groups. Mice with 5/6 nephrectomy exhibit albuminuria, progressive decline in kidney function and glomerular and tubulointerstitial fibrosis^42,43^. We observed an improved glomerular filtration rate (GFR) in CKD mice treated with the miR299a-5p anti-miR compared to mice treated with the control LNA, suggesting that miR299a-5p is protective against the decline in kidney function in CKD (Fig. 6C). Similarly, a reduction in albuminuria was observed with miR299a-5p anti-miR treatment (Fig. 6D). Lastly, these mice were also protected against the development of glomerulosclerosis and tubulointerstitial fibrosis as seen by decreased accumulation of collagens assessed using trichrome (Fig. 7A) and picrosirius red (PSR) staining (Fig. 7B), as well as decreased deposition of the extracellular matrix protein fibronectin, assessed by immunohistochemistry (Fig. 7C) and immunoblotting (Fig. 7D). Upregulation of the profibrotic cytokine CTGF was also inhibited (Fig. 7D). These changes were also associated with decreased expression of pSmad3 (Fig. 7E).

miR299a-5p has been shown to play a critical role in promoting cell growth and proliferation in cancer settings. Thus, we further determined whether miR299a-5p is involved in the regulation of renal cell proliferation in our model using immunostaining for Ki67. Our data show that miR299a-5p inhibition decreased proliferation in mice with CKD (Sup Fig. S3). Furthermore, to assess whether miR299a-5p inhibition exerts its therapeutic antifibrotic benefits via reduction of renal inflammation, we carried out renal CD3 and F4/80 immunohistochemistry to test for T-cell and macrophage infiltration, respectively. We observed that miR299a-5p inhibition does not significantly reduce the macrophage (Sup Fig. 4A) or T-cell infiltration seen in CKD kidneys (Sup Fig. 4B). Next, to determine which specific glomerular cell types might be protected in response to miR299a-5p inhibition, using immunostaining, we identified podocytes, mesangial cells and endothelial cells using the cell type-specific markers nephrin, α8-integrin and CD31, respectively. Here, we observed that CKD was associated with a decrease in glomerular endothelial cell and podocyte density as reported previously (Sup Fig. 5A,B)^44,45^. miR299a-5p inhibition significantly preserved podocytes (Sup Fig. 5A) and glomerular endothelial cells (Sup Fig. 5B). Attenuated nephrin (podocyte) staining was noted in the miR inhibition sham group. We are unable to determine the cause for this decrease. It may be that a greater number of mice need to be assessed, as no other parameters including albuminuria were worsened in this control group. Interestingly, glomerular mesangial cells, identified by α8-integrin immunofluorescence, were not affected by CKD or miR299a-5p inhibition. Overall, our results suggest that miR299a-5p inhibition in vivo ameliorates renal fibrosis and protects against the progression of CKD, associated with inhibition of Smad3 activation.

## Discussion

microRNAs (miRNAs) have been implicated in the regulation of a wide range of fundamental cellular processes and normal renal physiology. In a disease setting, these miRNAs have also been linked to progression of various diseases including kidney disease. Indeed, therapeutically targeting miRNAs using LNA anti-miR technology has become a highly investigated area with both overexpression and inhibition being used depending on the miRNA and its function in disease^23,27,28^. Here, we identified a novel miRNA, 299a-5p, that is important to the regulation of renal fibrosis. Its upregulation by TGFβ1 leads to attenuated production of the endogenous antifibrotic protein FST, enabling the profibrotic response. Importantly, inhibiting miR299a-5p attenuates renal fibrosis and protects against progression of CKD. miR299a-5p inhibition is thus a novel therapeutic approach for protecting against renal fibrosis in CKD.

Our previous studies identified an important role for caveolae and their marker protein cav-1 in regulating FST transcription, with cav-1 deletion significantly upregulating FST expression^14^. We now show that cav-1 also regulates FST post-transcriptionally, with the 3′UTR of FST significantly stabilized in the absence of cav-1. We thus sought to define if cav-1 regulation of miRNAs targeting the FST 3′UTR could explain our findings. miRNAs are short RNA sequences that bind to complimentary target sequences within the 3′UTR of a gene and act as silencers of gene expression or translational repressors^21^. Using bioinformatics tools, we identified numerous miRNAs that can target the FST 3′UTR. Here, we determined that cav-1 deficiency leads to downregulation of the expression of some of these FST-targeting miRNAs, with greatest downregulation seen for miR299a-5p. miR299a-5p is localized to the chromosome 14q32.31 cluster containing > 40 intergenic miRNAs, belonging to the miR154 family. This cluster is highly conserved between species^46,47^. Using transient miR299a-5p inhibition and overexpression, we validated a functional role for miR299a-5p in binding the 8-mer MRE within the 3′UTR of FST and in turn downregulating its transcript and protein expression. While numerous studies have identified key regulators of FST transcription, little is as yet know of regulation of its 3′UTR and no direct regulation by miRNAs has previously been described. One study in cancer cells identified non-miRNA-mediated regulation of the FST 3′UTR. Here, increased stability of this region, and hence of the FST mRNA, was mediated by reduced AUF1 binding to its AU-rich element in response to glucose deprivation. This protected cells from apoptosis^48^. Whether other miRNAs or 3′UTR binding proteins are regulated by cav-1 to affect FST expression remains to be determined.

Our results further showed that miR299a-5p is induced by the profibrotic cytokine TGFβ1. This was also shown for another member of this miR cluster, miR154, in lung fibroblasts. Interestingly, miR299a-5p was not upregulated in these cells, showing cell specificity in this response^35^. Furthermore, while Smad3 mediated miR-154 upregulation by TGFβ1, the mechanism by which TGFβ1 increases the expression of miR299a-5p in MC is not yet understood and needs to be further studied^35^.

While TGFβ1 induction of FST has been shown in differentiating bovine granulosa cells^49^, TGFβ1-mediated repression of FST has not as yet been described. In keeping with a suppressive role for miR299a-5p in FST 3′UTR stability and thus transcript expression, we observed that TGFβ1 inhibits FST expression. That this was mediated by the increase in miR299a-5p was confirmed by the ability of miR299a-5p inhibition to rescue this downregulation. Our data further showed the important role of FST downregulation by TGFβ1 in the profibrotic response to this cytokine. Indeed, in cav-1 KO cells which express high levels of FST, associated with suppressed miR299a-5p expression, their blunted TGFβ1 profibrotic response can be rescued by miR299a-5p overexpression, which as seen in our studies and by others, is associated and interlinked with Activin A secretion (activity) and Smad3 signaling^15,39^. These data support a major role for miR299a-5p in controlling the fibrotic response through its regulation of FST, likely mediated through a Smad3 dependent mechanism.

Recent studies have begun to implicate members of the miR154 family in fibrosis of various organs. The expression of miR299a-5p was found to be increased in mouse models of pulmonary and cardiac fibrosis and in fibrotic liver from patients with primary biliary cirrhosis^34–36^. Furthermore, in a model of myocardial infarction, increased miR299a-5p correlated inversely with cardiac function, and therapeutic inhibition of the family member miR-154 protected against cardiac dysfunction and fibrosis in a mouse model of pressure overload^34^. Until now, miR299a-5p or other miR154 family members have not been studied in MC and in the regulation of kidney pathology. Our data now show increased miR299a-5p in both glomeruli and tubules in a CKD model. We thus assessed whether inhibition of miR299a-5p in vivo using LNA anti-miR can inhibit renal fibrosis and protect against the progression of CKD. As expected, miR299a-5p inhibition increased the expression of FST in the kidneys of mice with CKD and this was associated with protection against renal fibrosis. More importantly, in vivo functional improvements were also seen with an increase in GFR and reduction in albuminuria in response to miR299a-5p inhibition. No changes in body weight were seen. Since elevated serum FST has previously been shown to induce muscle growth and increase body weight through its inhibition of myostatin^50–53^, this suggests minimal impact of miR inhibition on serum FST levels. This would be consistent with our previous observations of elevated FST in the kidneys of cav-1 KO mice^14^, with no significant elevation of serum FST levels (data not shown). Collectively, these results are in keeping with a major role for FST in the protection against renal fibrosis seen with miR299a-5p inhibition^15,40,41^.

Interestingly, other FST neutralization targets in addition to Activin A, such as myostatin, have also been shown to promote fibrosis in tissues such as skeletal muscle via a Smad3 dependent mechanism^54^. Efforts are being made to establish the role of myostatin and potential benefits of its inhibition in CKD-associated muscle wasting^55^. The role of myostatin in the kidneys in promoting fibrosis has not as yet been studied. Whether therapeutic FST overexpression or miR299a-5p inhibition protects against renal fibrosis through the blockade of these alternative FST neutralization targets needs to be more thoroughly examined in future studies.

Alternatively, FST expression/miR299a-5p inhibition may also reduce renal fibrosis through epigenetically regulating histone deacetylase (HDAC)-mediated expression of profibrotic proteins. HDACs induce deacetylation of proteins, which plays a critical role in the modulation of physiological and pathological gene expression. HDAC inhibition has been shown to block the progression of renal fibrosis in several animal models^56^. While FST was shown to be an essential mediator of HDAC inhibitor-induced increase in muscle size and satellite cell recruitment in vitro and in vivo*,* whether this pathway is important in regulating kidney fibrosis is not known. Furthermore, whether increased kidney FST expression, achieved by exogenous treatment or miR299a-5p inhibition, may potentiate therapeutic antifibrotic effects of HDAC inhibition also remains to be determined^57^.

In our study, we also examined the effects of miR299a-5p inhibition on several processes that contribute to the development of kidney fibrosis. Our studies did not show any effect on macrophage or T-cell infiltration, contrasting with the reduction of inflammation seen with FST in a model of cisplatin-induced acute kidney injury^58^. However, consistent with a critical role for miR299a-5p in promoting cell growth and proliferation in acute promyelocytic leukemia^59^, our data do show suppression of proliferation with miR299a-5p inhibition in CKD. Furthermore, our data also suggest that miR299a-5p inhibition protects against glomerular podocyte and endothelial cell loss associated with CKD. Interestingly, Smad3 signaling has been shown to be associated with high glucose-induced podocyte injury and apoptosis^60^. Thus, our data suggest that miR299-a5p inhibition, resulting in Smad3 pathway inhibition, may also protect against renal damage through the preservation of glomerular podocytes and endothelial cells.

Increasing renal FST expression is a promising antifibrotic treatment strategy^14,15,41,61^. We have previously shown that treatment with FST protects against CKD in 5/6 nephrectomized mice and in diabetic kidney disease^15,40^. While we observed increased renal FST by immunohistochemistry in treated mice, dosing was fairly frequent (daily or every other day), and the longer-term effects of FST tissue accumulation are unclear. Furthermore, we also observed that higher doses of FST led to reduced efficacy in renal protection, potentially through excess reduction of oxidative species which are required for normal cellular signaling^40^. Our data demonstrating that endogenously expressed FST can be augmented in CKD through miR299a-5p inhibition to provide renal protection provide an important alternative to increasing renal FST without leading to supraphysiologic levels.

## Methods

### Cell culture

Primary mouse mesangial cells (MCs) were isolated from cav-1 wild-type (WT) and cav-1 KO B6129SF1/J mice (Jackson Laboratory), as described previously^14^. MCs were grown in Dulbecco’s modified Eagle’s medium (DMEM) supplemented with 20% fetal bovine serum (Invitrogen), penicillin (100 μg/ml) and streptomycin (100 μg/ml) at 37 °C in 95% O_2_, 5% CO_2_. Passages 7–14 were used. MCs were serum deprived in 0.5% FBS 24 h prior to treatment unless otherwise stated. Drugs and reagents used in the study are provided in Supplementary table 1.

### Transfection

Transient expression of plasmids was achieved using electroporation with the ECM 830 Square Wave Electroporation System (Harvard Bioscience). Briefly, MCs resuspended in electroporation buffer containing the appropriate plasmids (0.5 μg luciferase plasmid with 0.05 μg β-galactosidase or 10 μg protein expression plasmid) were electroporated using a single square pulse set at 200 V for 35 ms. Effective transfection of miR inhibitor and overexpression clones was confirmed by observing mCherry (ex550 nm/em620 nm) and GFP (ex490 nm/em525 nm) immunofluorescence, respectively (EVOS FL Cell Imaging System, Thermo Fisher Scientific). SiRNA-mediated (50 nM) knockdown was achieved using RNAiMAX (Thermo Fisher Scientific) as per the manufacturer’s recommendation. MCs were serum deprived 24 h following transfection prior to treatment and harvest. Plasmids and siRNA used in the study are provided in Supplementary tables 2 and 3.

### Luciferase assay

MC lysis was achieved using Reporter Lysis Buffer (Promega) as per the manufacturer’s recommendation. Luciferase (luc) activity was measured on clarified cell lysate using the Luciferase Assay System (Promega) with a luminometer (Junior LB 9509, Berthold). β-galactosidase activity, used to normalize for transfection efficiency, was measured in clarified cell lysates using the β-Galactosidase Enzyme Assay System (Promega) with a plate reader absorbance set at 420 nm (SpectraMax Plus 384 Microplate Reader, Molecular Devices).

### Protein analysis

MC cell lysis and protein extraction were carried out as described previously^62^. Briefly, cell lysates were centrifuged (15,000 rpm, 10 min, 4 °C), supernatant was collected and protein concentration quantified. Cell protein lysates (10 μg-50 μg) were separated on SDS-PAGE for subsequent immunoblotting. Tissue samples were homogenized using a bead mill homogenizer (Bead Ruptor Elite) and 1.4 mm ceramic beads (Lysing Matrix D, MP Biomedicals), then centrifuged (15,000 rpm, 10 min, 4 °C), supernatant collected and protein concentration quantified. Tissue protein lysates (50 μg) were separated on SDS-PAGE for subsequent immunoblotting. Primary antibodies used in the study are provided in Supplementary table 4. Full-length blots are provided in Sup Figs. S6, S7, S8 and S9.

### Activin A ELISA

Secreted activin A was quantified from clarified (15,000 rpm, 10 min, 4 °C) MC culture media using the activin A Quantikine ELISA Kit (R&D Systems).

### mRNA and miRNA extraction and quantitative-real time PCR

RNA from MCs was extracted using Ribozol RNA Extraction Reagent (Amresco) as per the manufacturer’s recommendation, with 1 μg of RNA reverse transcribed into cDNA using qScript cDNA SuperMix Reagent (Quanta Biosciences). miRNA-enriched cDNA was generated using the qScript microRNA Quantification System (Quanta Biosciences). Quantitative real-time PCR was carried out using the Power SYBR Green PCR Master Mix (Thermo Fisher Scientific) on the Applied Biosystems ViiA 7 Real-Time PCR System (Thermo Fisher Scientific). mRNA and miRNA expression and fold changes were calculated using the ΔΔC_T_ method, where 18S was used as a control for mRNA and U6 snRNA as a control for miRNA. Primers sequences used in the study are provided in Supplementary table 5.

### Cloning

miR299a-5p regulatory element (MRE) luc was generated in order to measure miR299a-5p activity. Briefly, a 5′-phosphorylated oligonucleotide encoding a 67-bp region within the FST 3′UTR that includes the mir299a-5p 8mer MRE was synthesized. No other miRNA MRE completely localized and/or overlapped within this region. This oligonucleotide was inserted into a XbaI digested pGL3 vector. All sequences synthesized for cloning are listed in Supplementary table 6. All generated constructs were confirmed by sequencing (Mobix Lab, McMaster University).

### miRNA in situ hybridization (ISH)

6 μm FFPE kidney sections were treated with proteinase K (10 min, 37 °C), followed by fixation in 4% PFA (10 min, RT). Subsequently sections were incubated in hybridization buffer (1 hr, 52 °C) and then incubated with DIG labeled miRCURY LNA anti-miR detection probes targeting miR299a and U6 probes (18 h, 52 °C). Probe details are provided in Supplementary table 2. Stringency washes were carried out exactly as described^63^. Sections were blocked in 1× Casein Solution (Vector labs) (1 h, RT) and incubated with anti-Digoxigenin-AP Fab fragment (1:100, 18 h, 4 °C). Chromogenic reaction was carried out using NBT/BCIP (dark, RT, 4 h–6 h) (Vector labs). Slides were then mounted with Vectamount (Vector labs) and examined using light microscopy (BX41 Olympus).

### Animal studies

All animal studies were approved by the McMaster University Animal Research Ethics Board (animal ethics protocol number: 16-07-27) and carried out in accordance with the principles of laboratory animal care placed by McMaster University and Canadian Council on Animal Care guidelines. Male CD1 mice were obtained from Charles River Laboratories. CKD was achieved using the 5/6 nephrectomy renal mass reduction model (Fig. 5E). Briefly, at 6–7 weeks, anesthetized mice underwent resection of the upper and lower poles of the left kidney. After a 1-week recovery period, anesthetized mice underwent a right nephrectomy. Sham mice were anesthetized and the kidney manipulated without resection. Resected kidney weights were divided by the nephrectomized right kidney weight (Nx ratio) and mice were placed into 4 groups, with the Nx groups containing mice with roughly equal Nx ratios: Sham-con-anti-miR (n = 5), Sham-mir299a-5p-anti-miR (n = 5), 5/6 Nx-con- anti-miR (n = 17), and 5/6 Nx-miR299a-5p-anti-miR (n = 17). Mice were injected subcutaneously (SC) once 24 h before right nephrectomy, and then with 2 mg/kg SC con-LNA or miR299a-5p anti-miR twice a week for study duration. At study endpoint (week 9), urine was collected and albumin-to-creatinine ratio measured according to manufacturer’s instructions (Albuwell M, Exocell for urine albumin and Crystal Chem for creatinine). Glomerular filtration rate (GFR) was assessed in conscious mice by measuring the clearance of fluorescein isothiocyanate (FITC)-labeled sinistrin (Fresenius Kabi Linz, Austria). Briefly, a 5% FITC-sinistrin solution was injected retro-orbitally, after which blood was collected from the saphenous vein at 7, 15, 30, 60, 90 and 120 min. Plasma fluorescence was assessed using a fluorometer (Gemini EM, Molecular Devices) at 485 nm excitation and 538 nm emission. Following GFR assessment, mice were perfused with cold PBS. Kidney portions were snap-frozen in liquid nitrogen (immunoblotting), stored in optimal cutting temperature (O.C.T) compound for immunofluorescence (IF) staining or fixed in formalin for immunohistochemistry (IHC) staining. Trichrome (Sigma) and PSR (Sigma) staining was done on 4 μm FFPE kidney sections following deparaffinization, according to manufacturer’s instructions. Heat-induced epitope retrieval was carried out on 4 μm FFPE kidney sections prior to IHC staining of fibronectin, FST, CD31 and CD3. Protease K digestion (40 µg/ml, 5 min) was carried out prior to IHC staining of pSmad3. F4/80 staining was done by the McMaster Histology Facility. 4% paraformaldehyde fixation (15 min, RT) was carried out on 6 μm O.C.T embedded frozen kidney sections prior to IF staining of nephrin and α8-integrin, with DAPI being used as a counterstain to delineate nuclei. Primary antibodies used in the study are provided in Supplementary table 4. Images were quantified by measuring the percentage of total signal positive area examined under transmitted light (or polarized light for PSR staining) or fluorescence (ex: 578 nm, em: 603 nm), using ImageJ or MetaMorph, either for the entire kidney cross section, or separately for glomerular and tubular interstitial compartments. All micrographs were captured at 200× and 400× magnification using the BX41 Olympus microscope (light/polarized microscopy) or the Olympus 1X81 microscope (fluorescence microscopy).

### Statistical analysis

Statistical analyses were performed using GraphPad Prism 6. A Student’s *t*-test or one-way ANOVA were used to determine statistical significance between two or more groups of data, respectively. Post hoc significance of pairwise comparisons was assessed using Tukey’s HSD. A p-value < 0.05 (two-tailed) was considered significant. Data are presented as mean ± SEM. The number of experimental repetitions (*n*) is indicated in the figure captions.

## Supplementary Information

Supplementary Information.
