# Supplementary material for: miR299a-5p promotes renal fibrosis by suppressing the antifibrotic actions of follistatin
Source: Sci Rep. 2021 Jan 8;11:88. doi: 10.1038/s41598-020-80199-z (PMC7794215; doi:10.1038/s41598-020-80199-z)
Supplement: Supplementary file 1 — Supplementary Information. [file 41598_2020_80199_MOESM1_ESM.pdf]

miR299a-5p Promotes Renal Fibrosis by Suppressing the Antifibrotic Actions of Follistatin

Neel Mehta[1], Renzhong Li[1], Dan Zhang[1], Asfia Soomro[1], Juehua He[1], Ivan Zhang[1], Melissa MacDonald[1], Bo Gao[1], Joan C. Krepinsky[1]

mehtan8@mcmaster.ca, lirenz@mcmaster.ca, dandantotoro@gmail.com, soomroa@mcmaster.ca, juehuahe@hotmail.com, ivan99zhang@hotmail.com, melmacdo@stjosham.on.ca, gaolinbo@hotmail.com, krepinj@mcmaster.ca

[1] Division of Nephrology, Department of Medicine, McMaster University, Hamilton, Canada

Correspondence to:

Dr. Joan C. Krepinsky
St. Joseph's Hospital
50 Charlton Ave East
Rm T3311
Hamilton, ON
L8N 4A6, Canada
Tel: 1-905-522-1155 x34991
Fax: 1-905-540-6589
E-mail: krepinj@mcmaster.ca

Running Title: miR299a-5p regulates renal fibrosis
Abstract word count: 202
Text word count: 3896 (excluding methods and figure legends)
Figure Count: 7 main, 9 supplemental
Supplemental Files: 2

Supplemental Figures

Figure S1a. Confirmation of the downregulation of FST by FST-targeting siRNA in MC (n=2).

Figure S1b. Validation of the FST H114 antibody using recombinant (rec.). To increase FST detection, cav-1 WT MC were treated with recombinant FST (0.1µg for 1h). Internalized (and endogenous) FST was detected by immunoblotting and compared to immunoblotting for recombinant FST alone (1µg). Multiple bands of the FST protein are observed around the expected size (~38-48kDa) in MC lysate due to cellular post-translational modifications. Recombinant FST (cell-free) probed with the FST H114 antibody shows an expected single band of around 40kDa.

Figure S2a. Overexpression of the miR299a-5p precursor increased miR-299a-5p levels, assessed using qRT-PCR (n=2).

Figure S2b. TGFβ1 (24h)-induced Activin A secretion in Cav-1 WT MC was repressed with miR299a-5p inhibition (n=3, *p<0.05).

Figure S2c. TGFβ1 (24h)-induced Activin A secretion in Cav-1 WT KO was augmented with miR299a-5p overexpression (n=3, *vs con inh-con, # vs con-inh- TGFβ1, p<0.05).

Figure S3. Renal cell proliferation was increased in CKD mice, as assessed by Ki67 immunohistochemistry. This was attenuated by miR299a-5p anti-miR administration (*vs Sham-Con-anti-miR, #vs 5/6 Nx-Con-anti-miR, scale bar = 200µm).

Figure S4a. CKD mice exhibited significant macrophage accumulation, as assessed by F4/80 immunohistochemistry, which was not affected by miR299a-5p anti-miR treatment (*vs Sham-Con-anti-miR, scale bar = 200µm).

Figure S4b. CKD mice exhibited significant CD3+ T lymphocyte infiltration, as assessed by CD3 immunohistochemistry, which was not affected by miR299a-5p anti-miR treatment (*vs Sham-Con-anti-miR, scale bar = 200µm).

Figure S5a. CKD mice exhibited a significant loss in podocytes, as assessed by nephrin immunofluorescence, which was attenuated by miR299a-5p anti-miR treatment (*vs Sham-Con-anti-miR, #vs 5/6 Nx-Con-anti-miR, scale bar = 5µm).

Figure S5b. CKD mice exhibited a significant loss in glomerular endothelial cells, as assessed by CD31 immunohistochemistry, which was attenuated by miR299a-5p anti-miR treatment (*vs Sham-Con-anti-miR, #vs 5/6 Nx-Con-anti-miR, scale bar = 100µm).

Figure S5c. No change in glomerular mesangial cells was seen either in CKD or with miR299a-5p anti-miR treatment, as assessed by immunofluorescence for α8-integrin (scale bar = 5µm).

Figure S6. Original western blots for fig1A, fig S1

Figure S7. Original western blots for fig 2D, 2F

Figure S8. Original western blots for fig 3D, 3E

Figure S9. Original western blots for fig 4A, 4B, 4C, 7D

Fig S1a

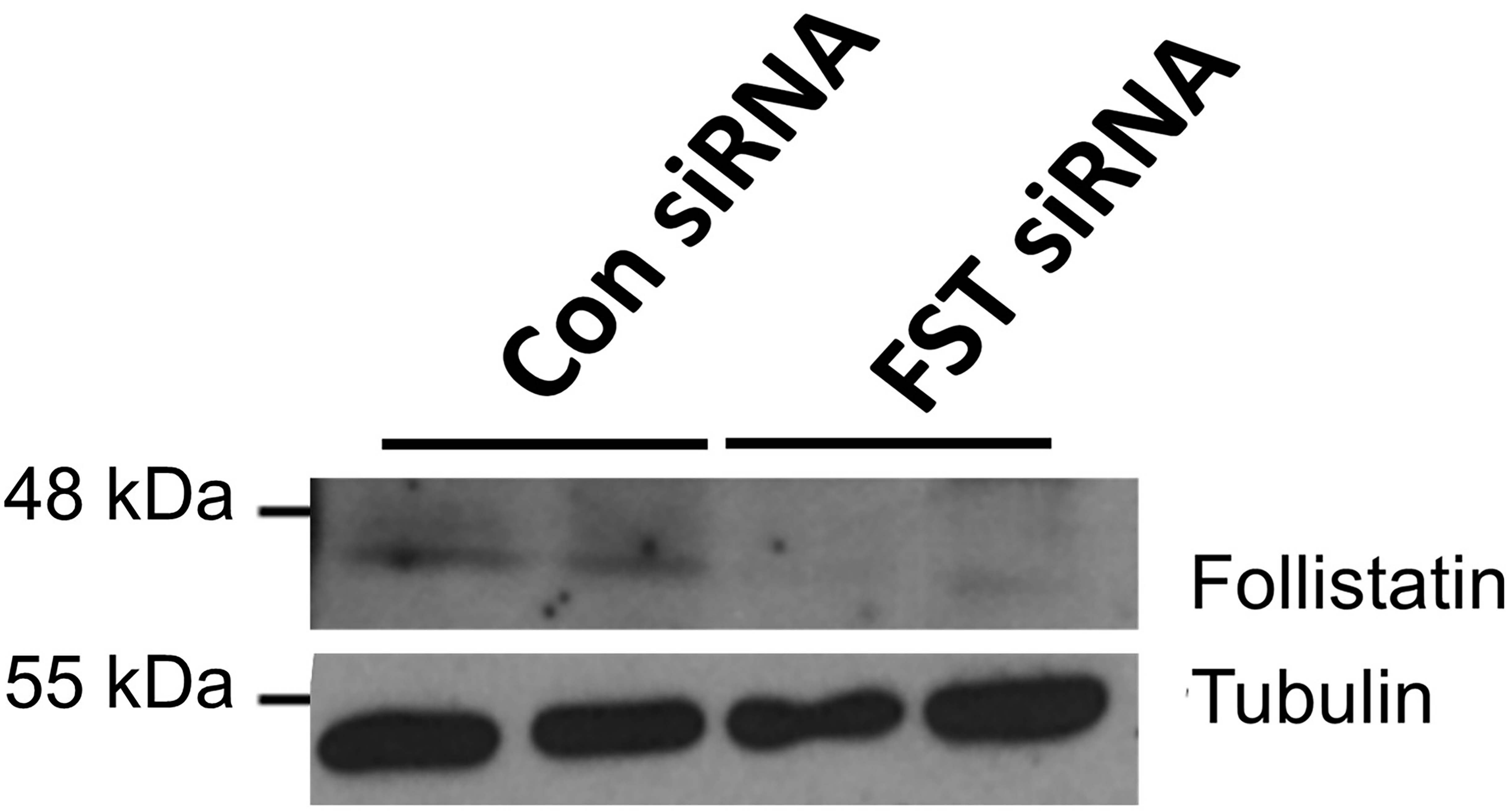

Fig S1b

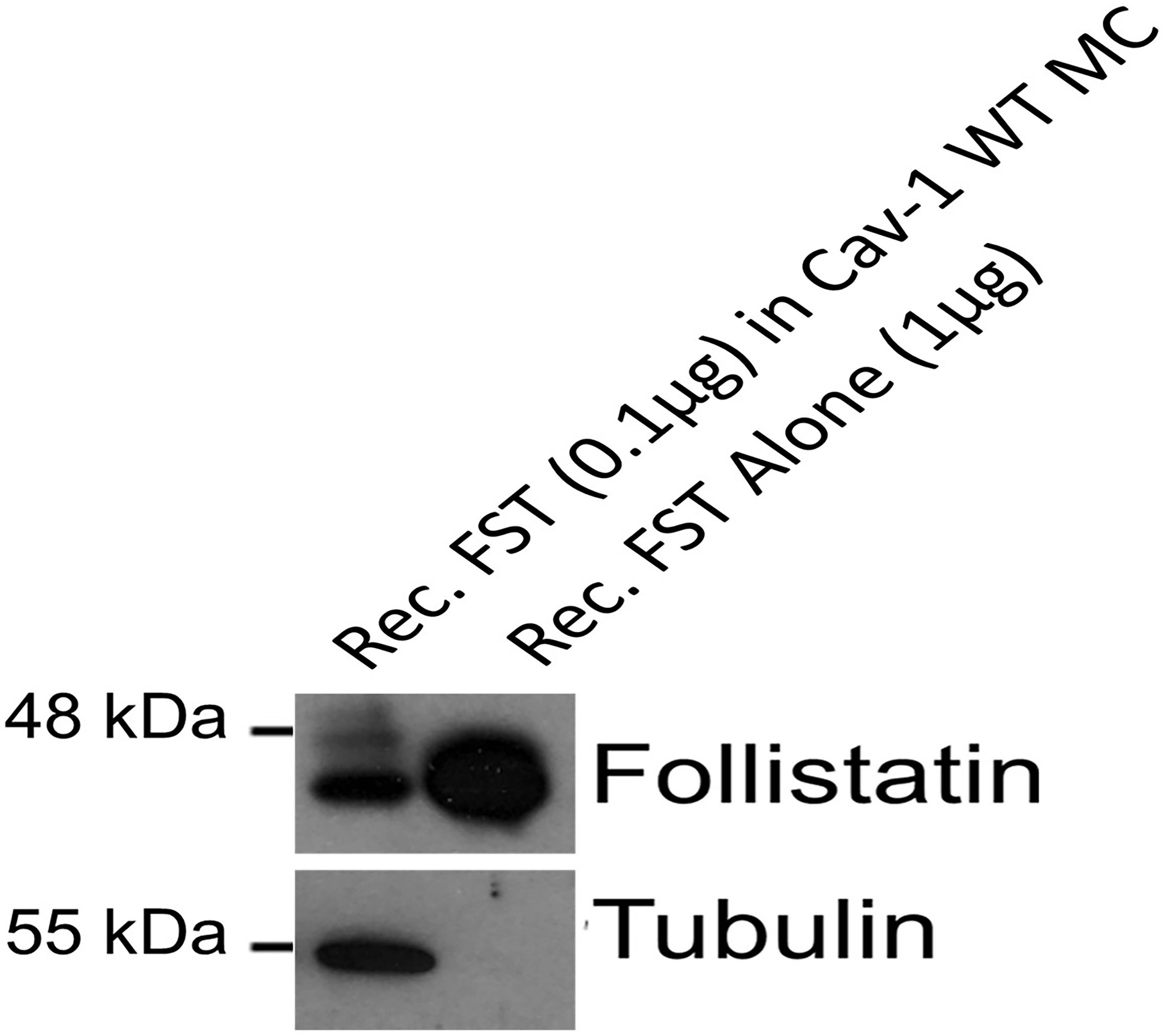

Fig S2a

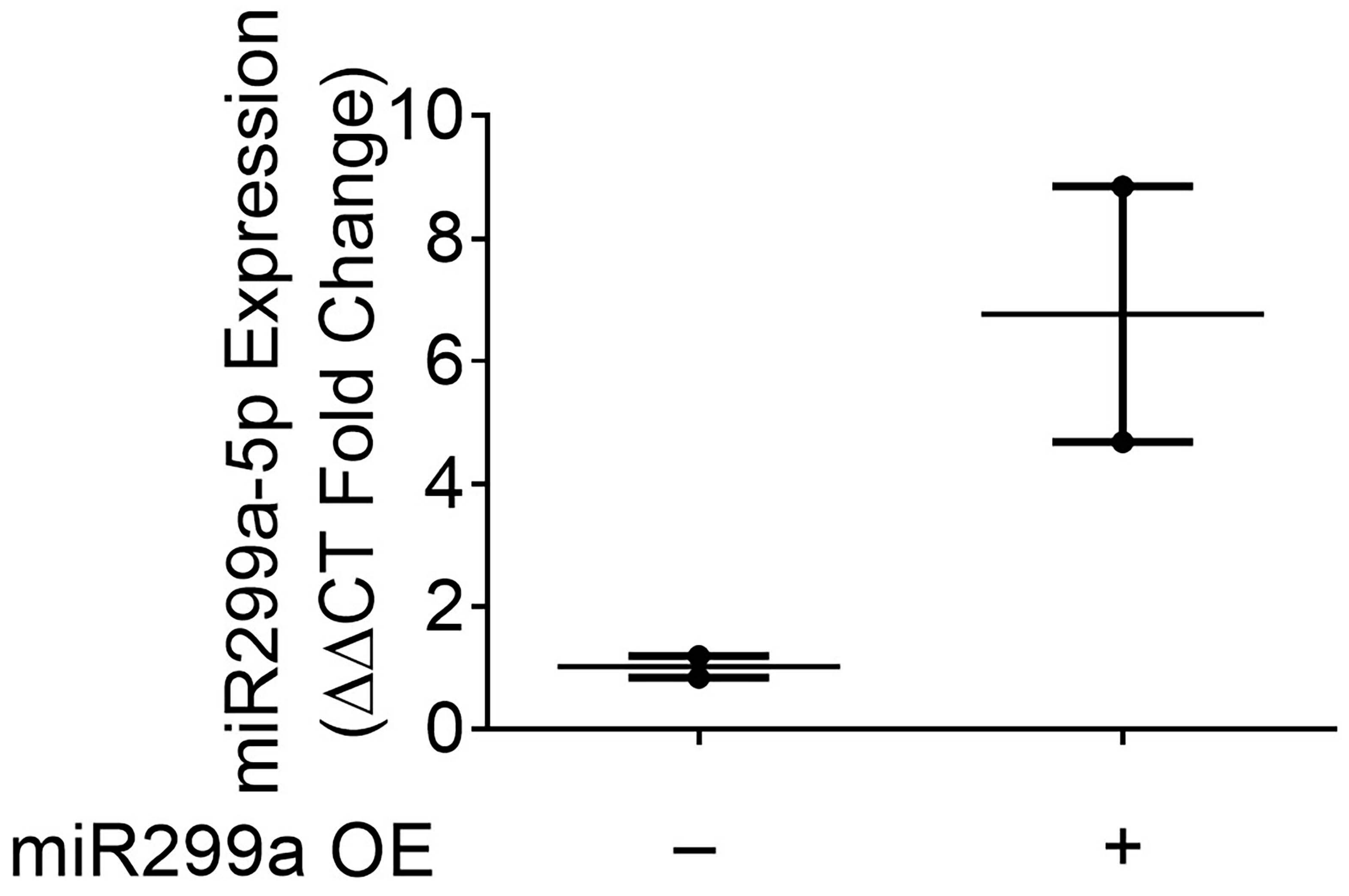

Fig S2b

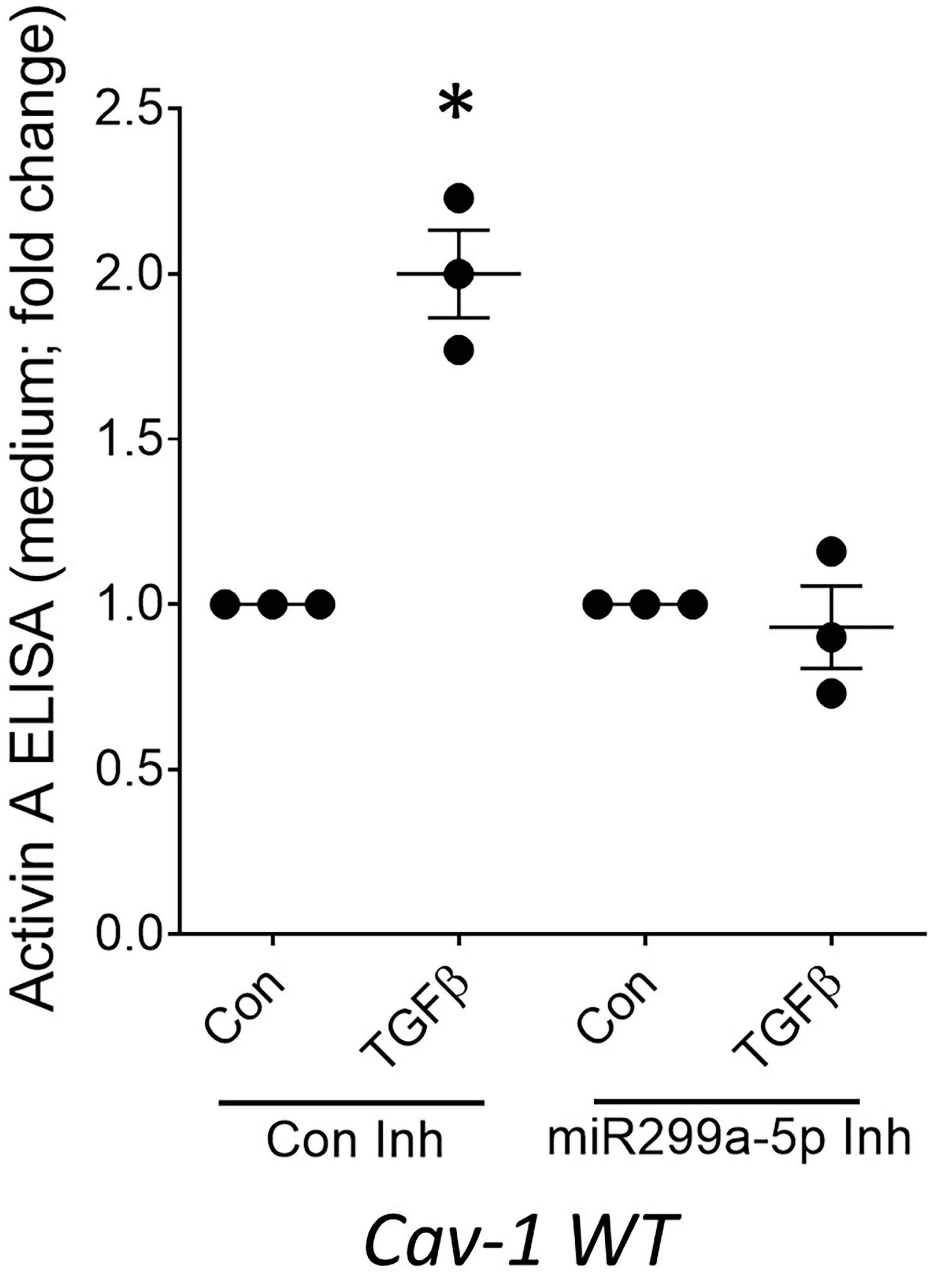

Fig S2c

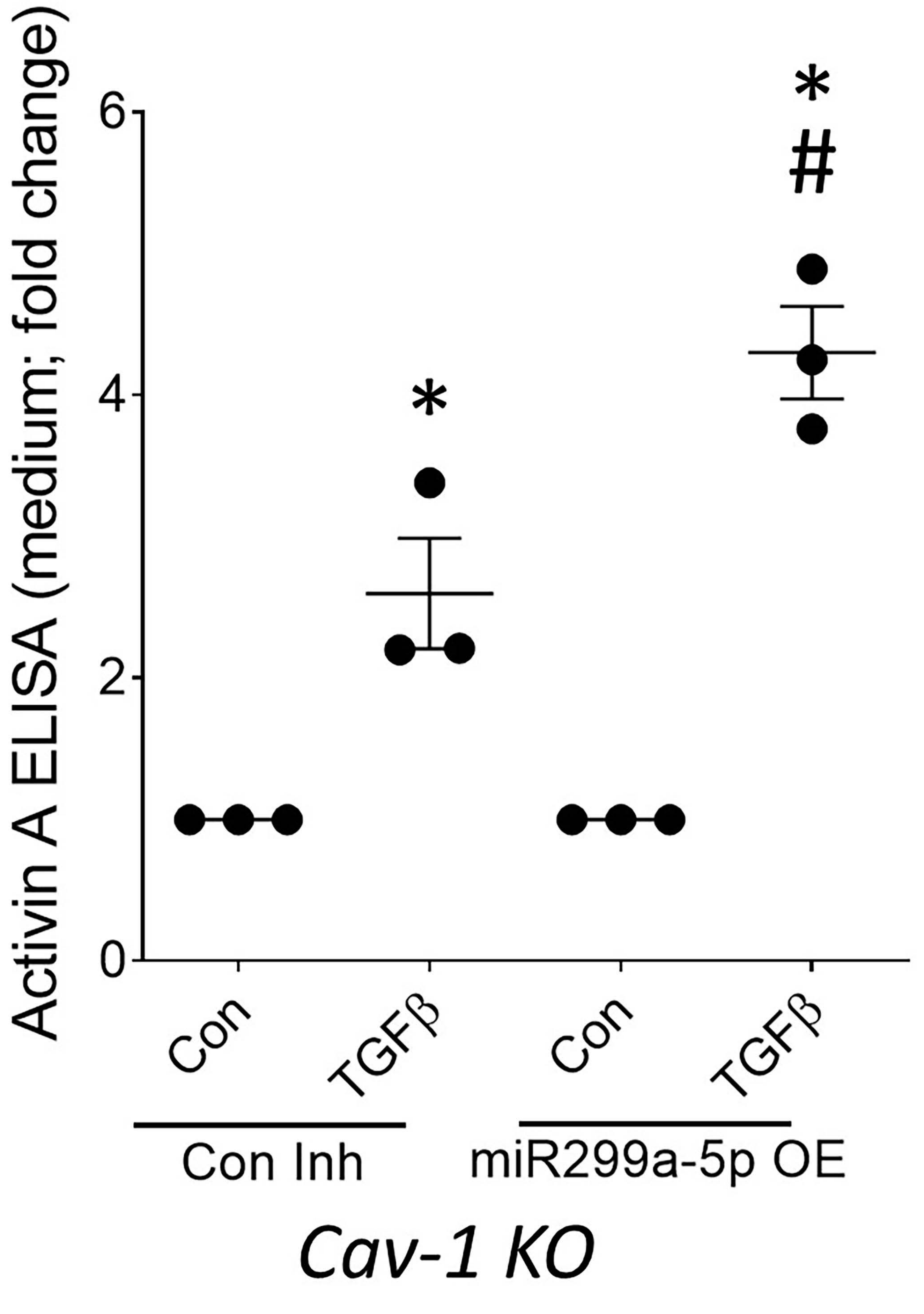

Fig S3

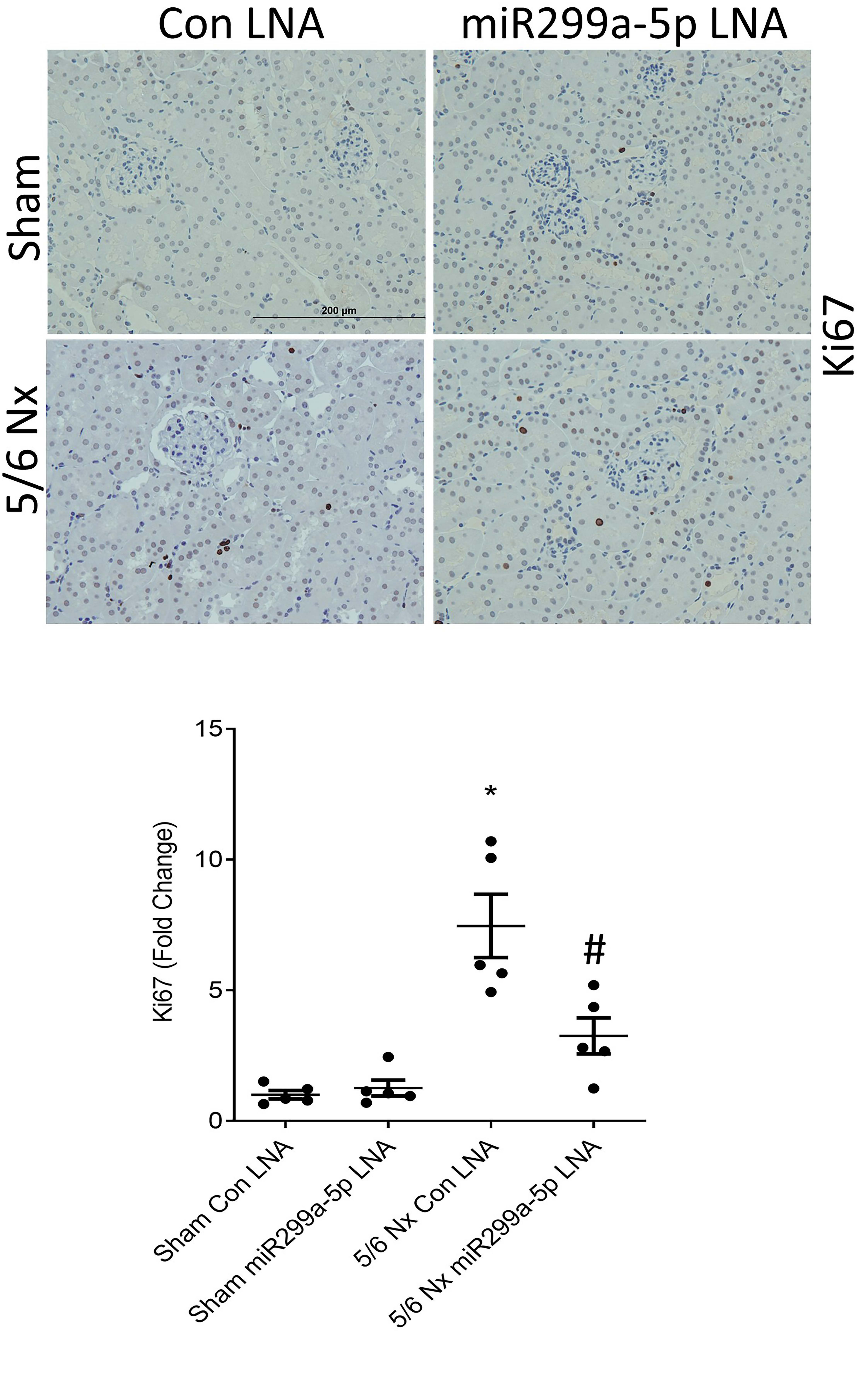
Con LNA
miR299a-5p LNA
Sham
5/6 Nx
Ki67
200 μm
15
10
5
0
Ki67 (Fold Change)
*
#
Sham Con LNA
Sham miR299a-5p LNA
5/6 Nx Con LNA
5/6 Nx miR299a-5p LNA

Fig S4a

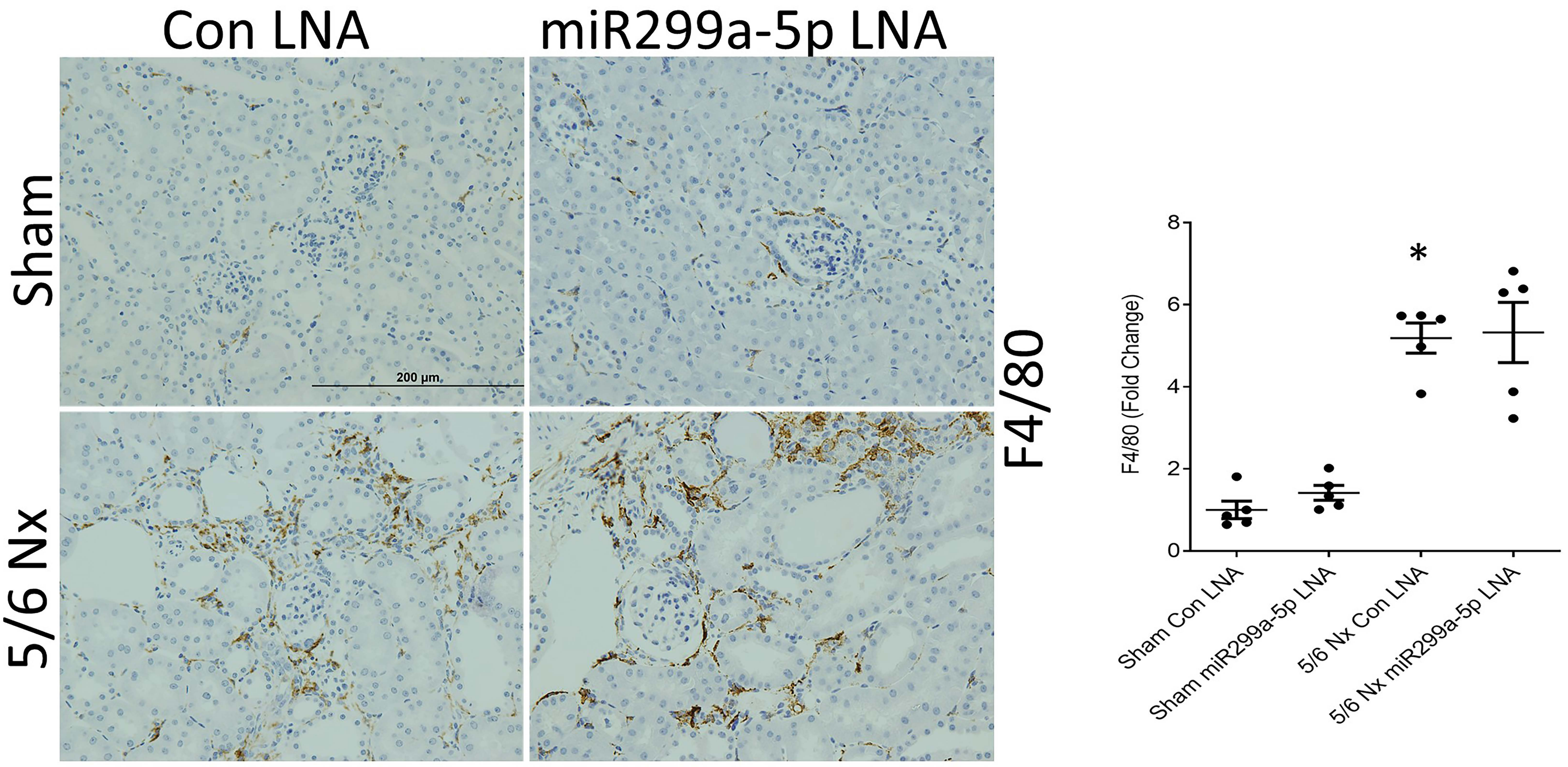

Fig S4b

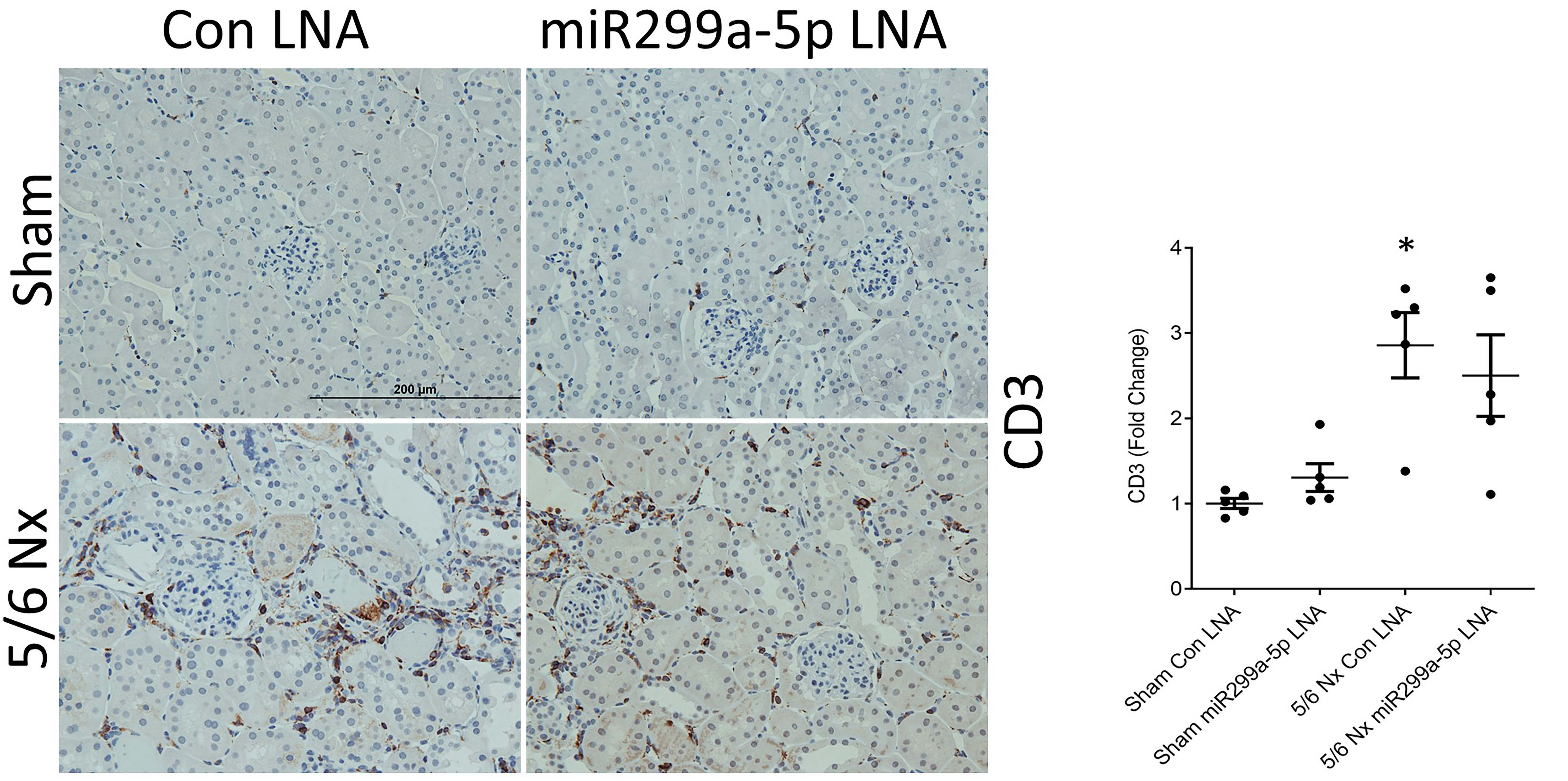

Fig S5a

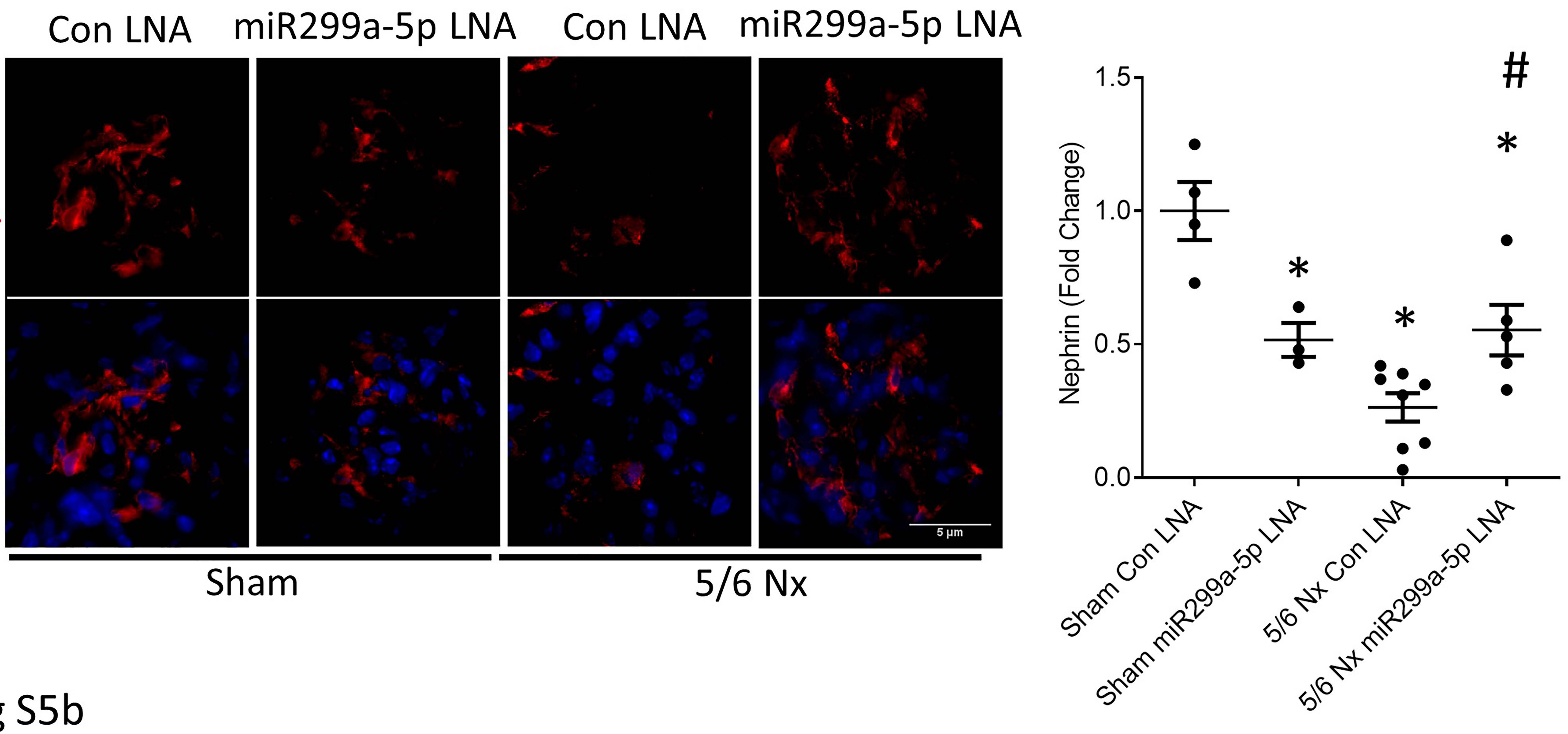

Fig S5b

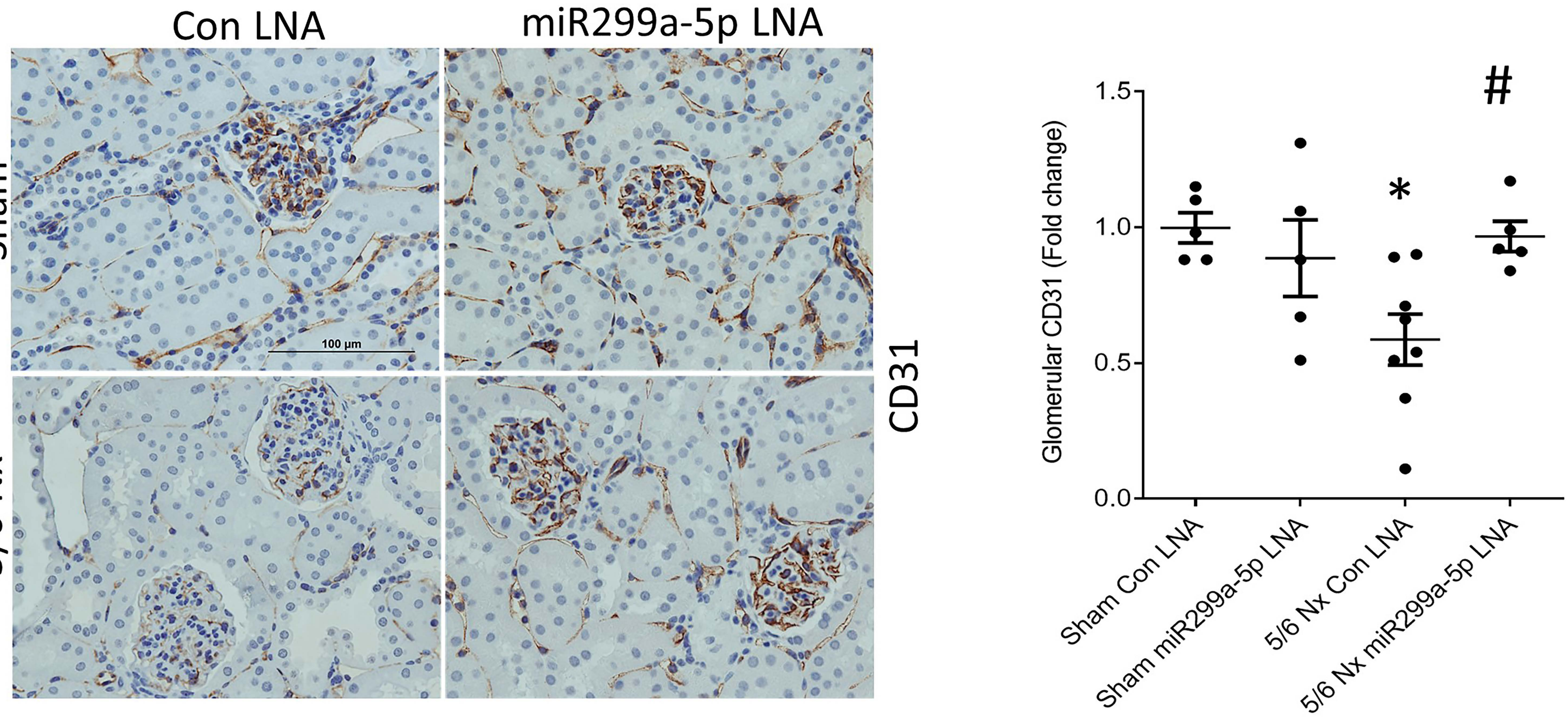

Fig S5c

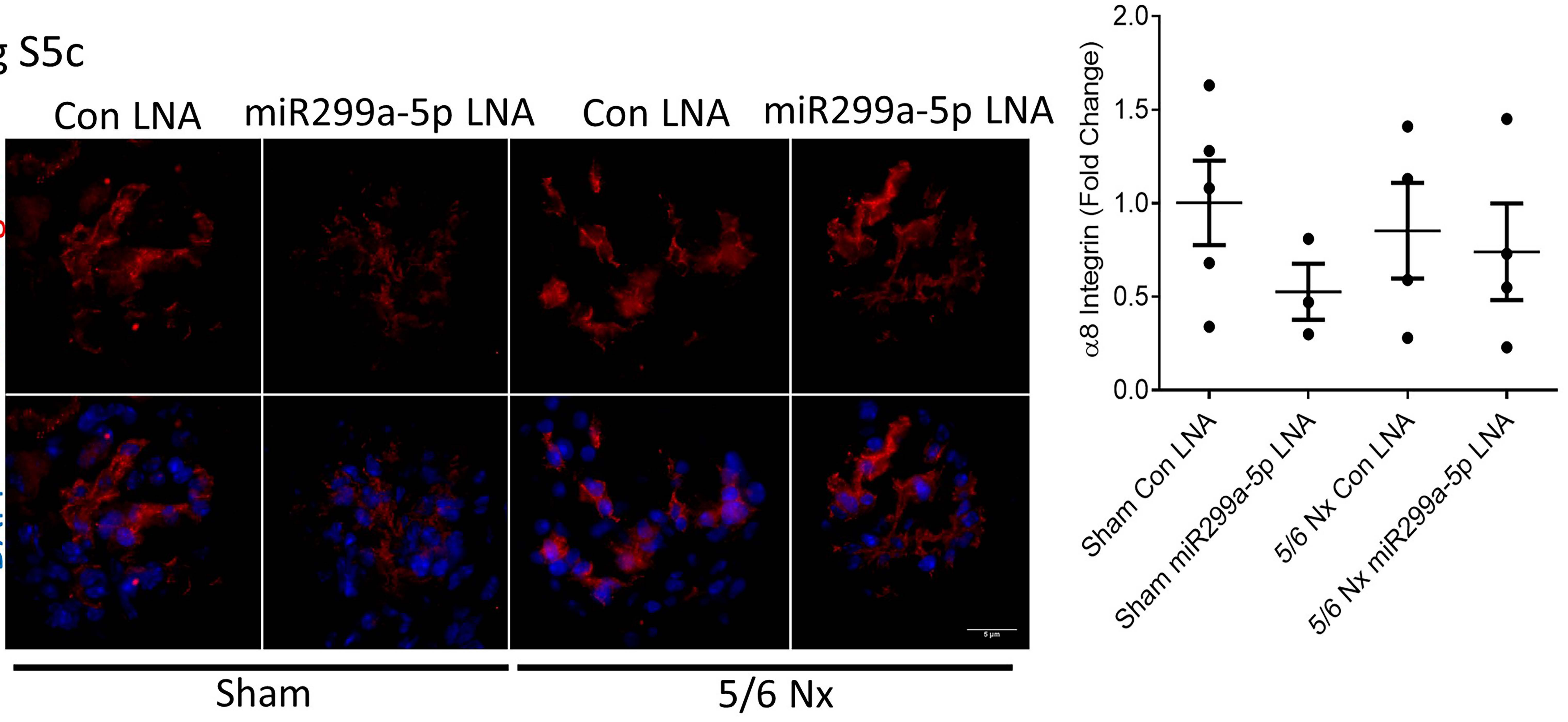

Fig S6

1A

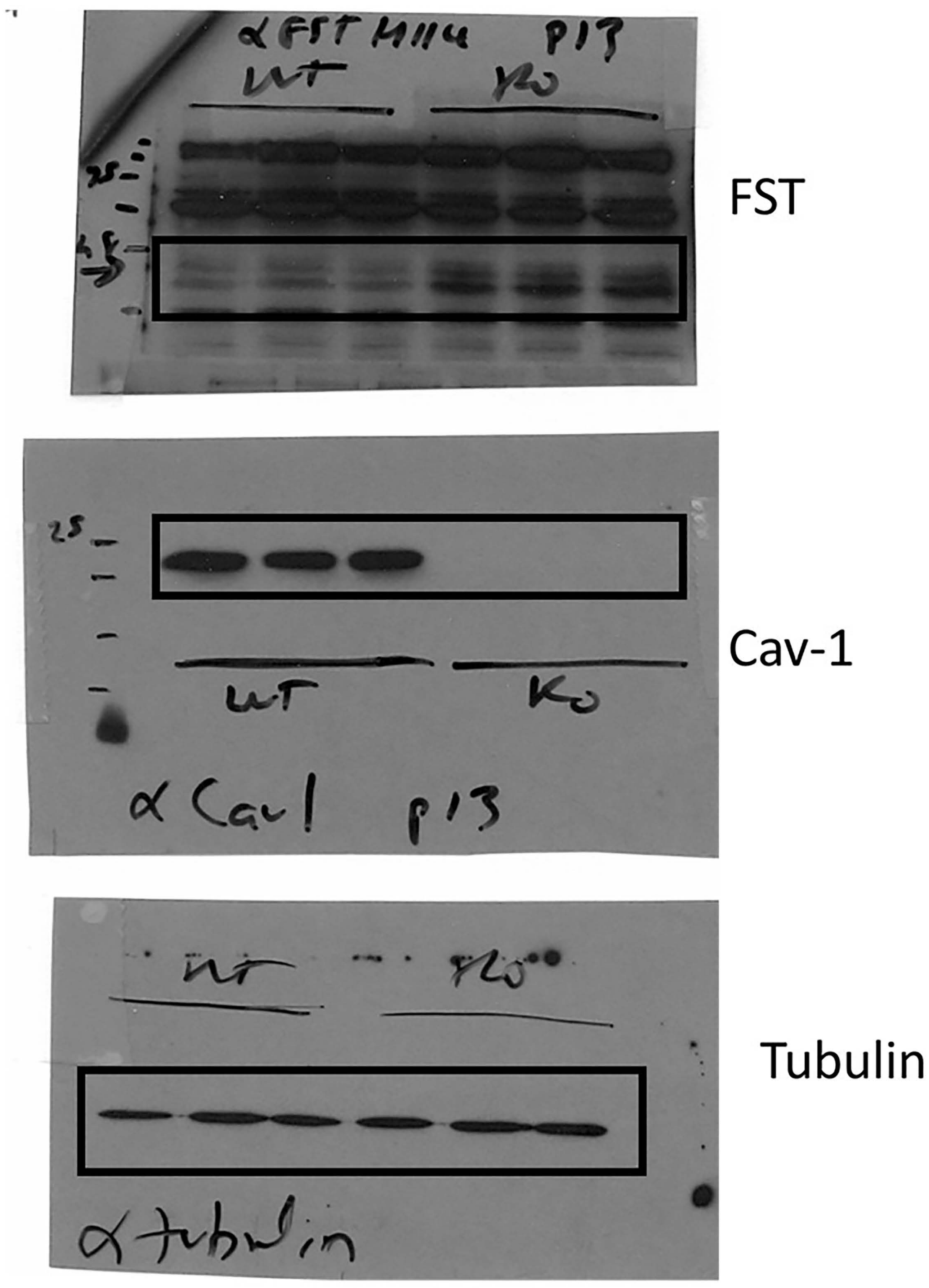

S1A

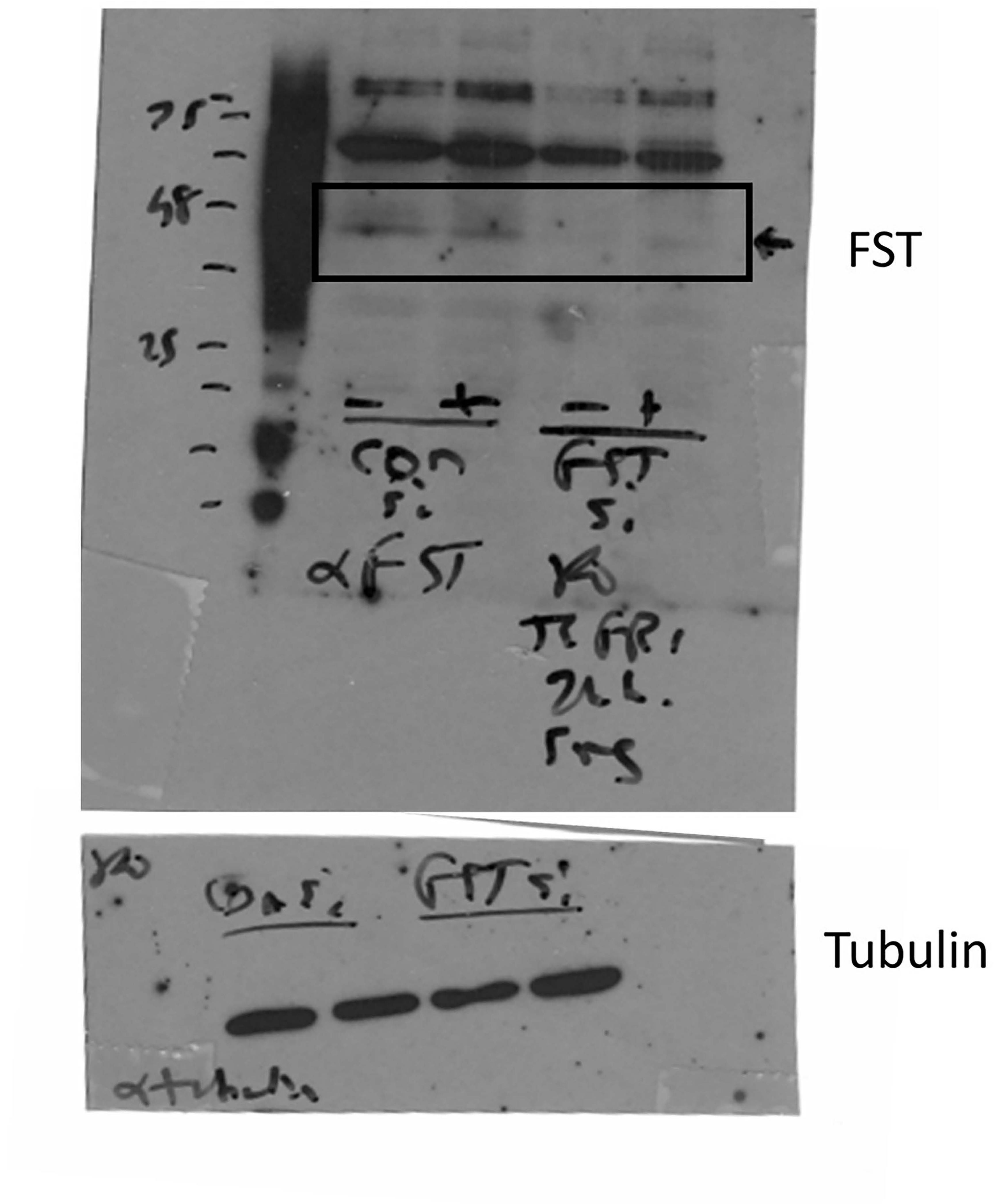

S1B

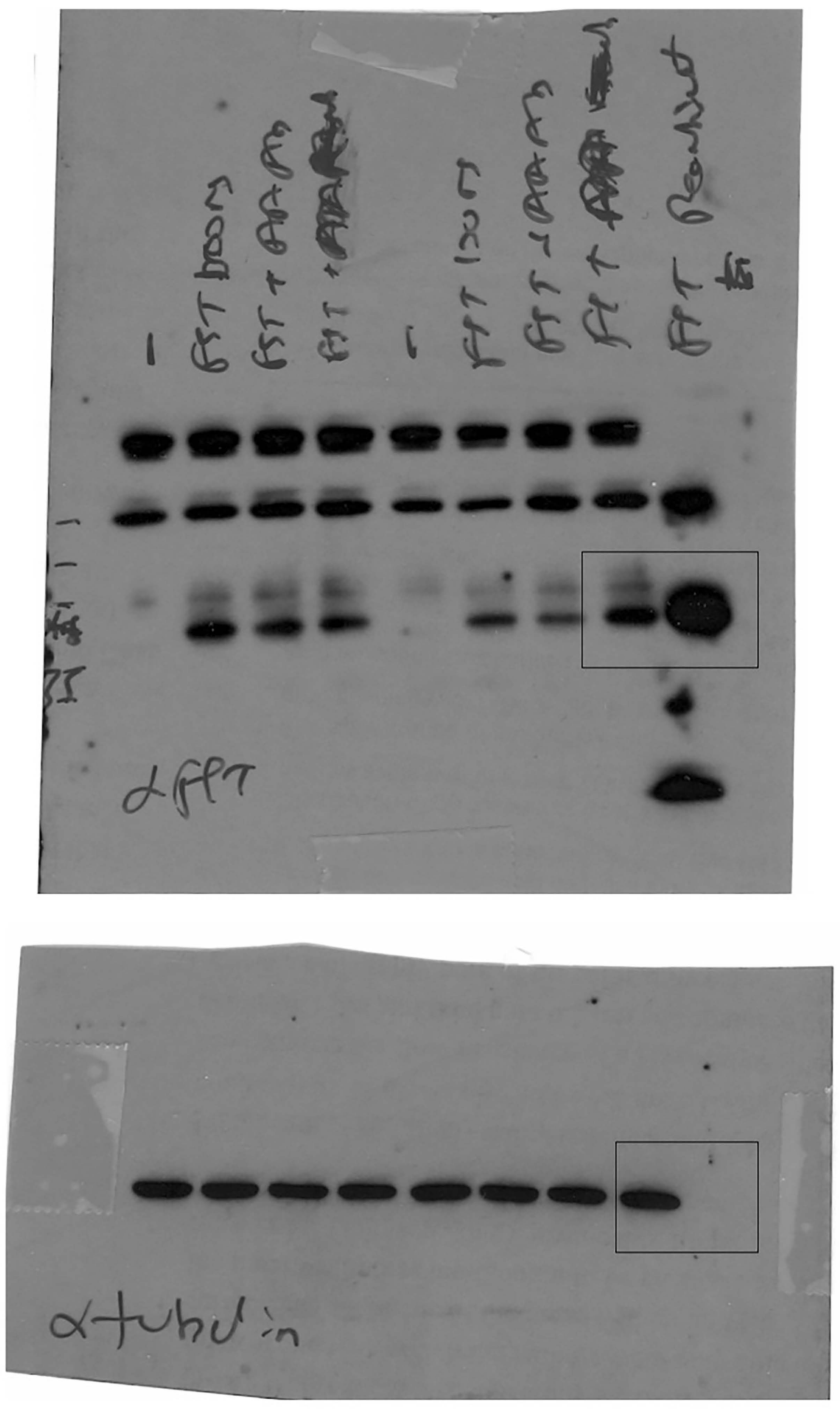

Fig S7

2D

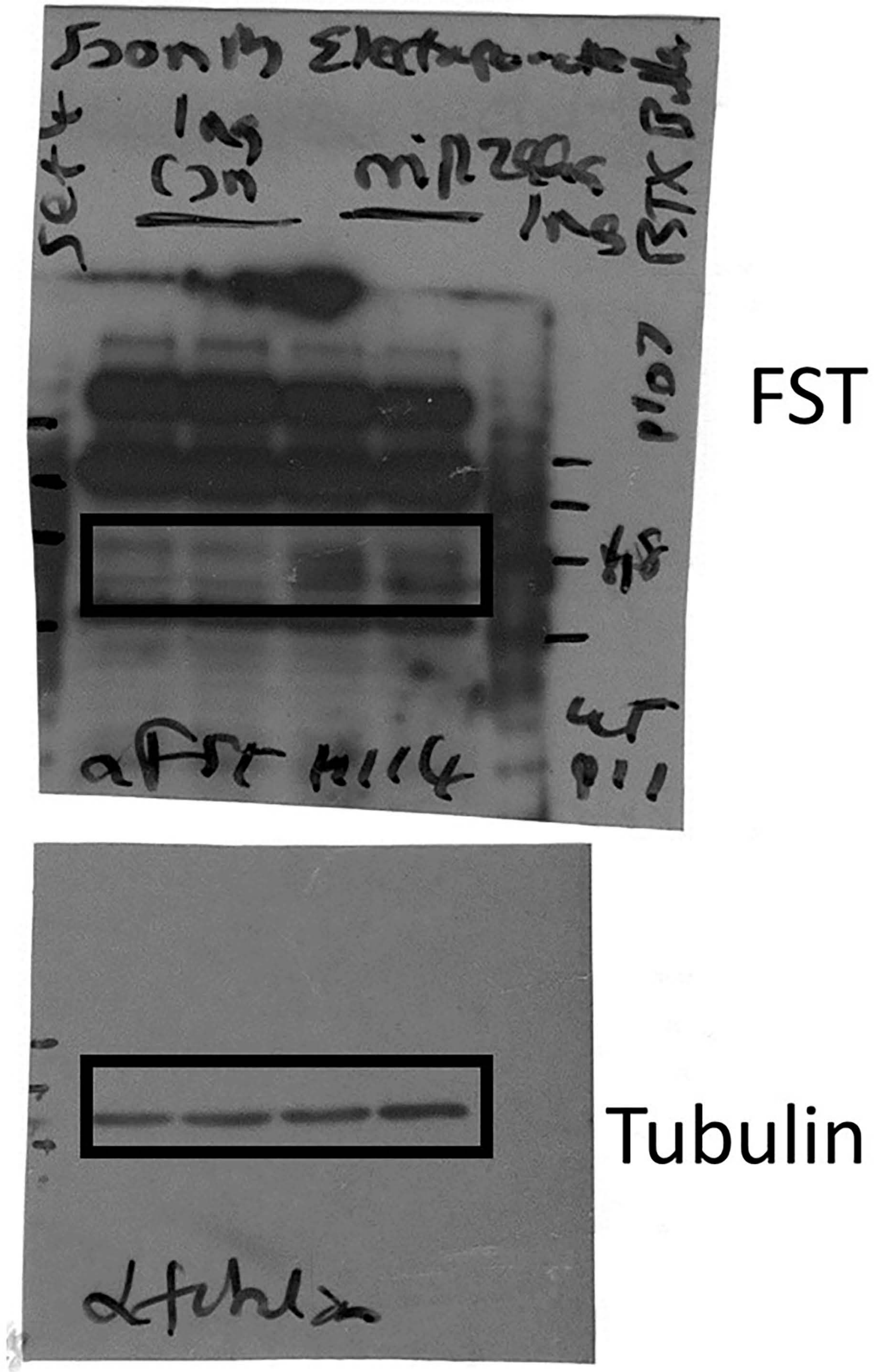

2F

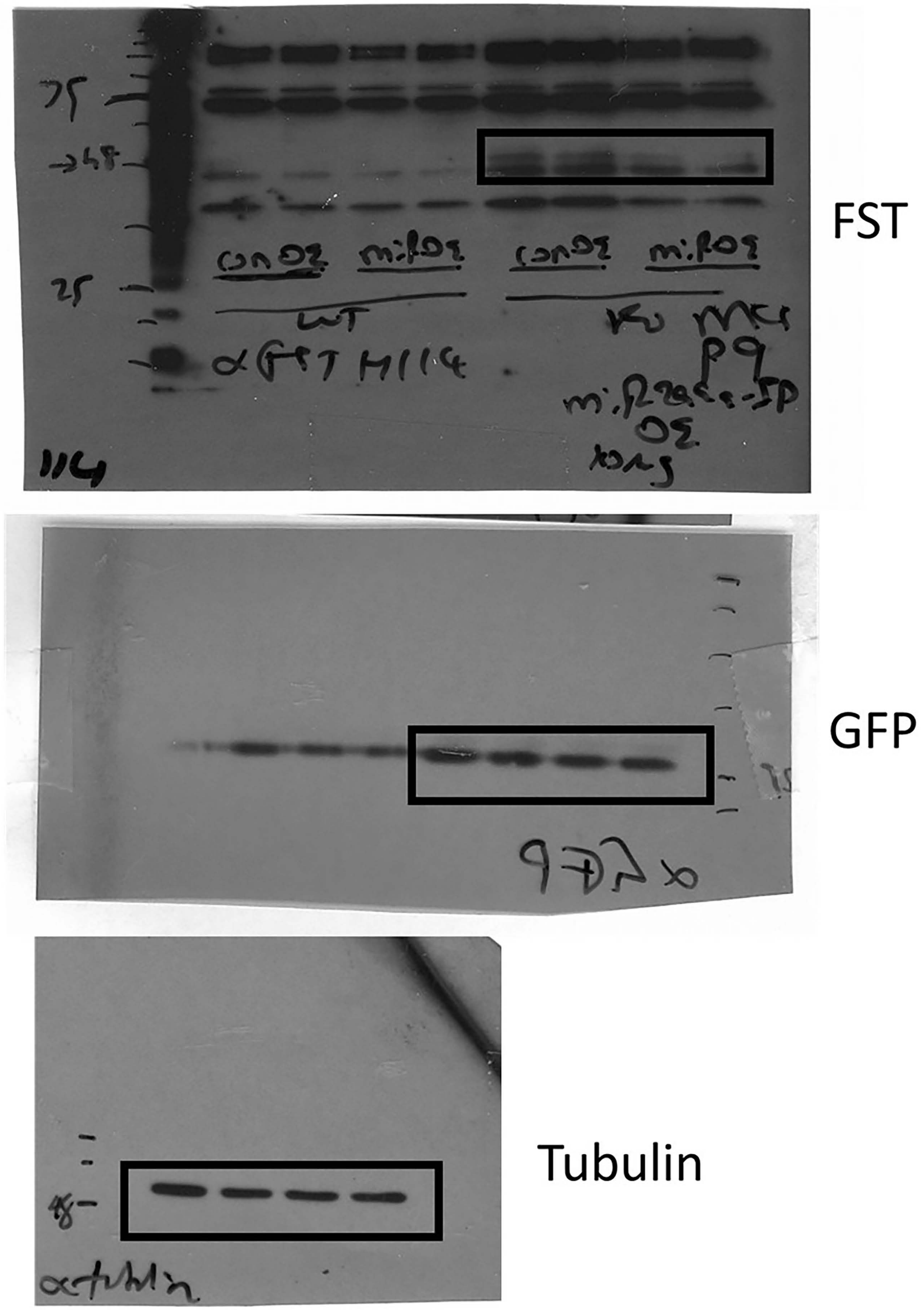

Fig S8

3D

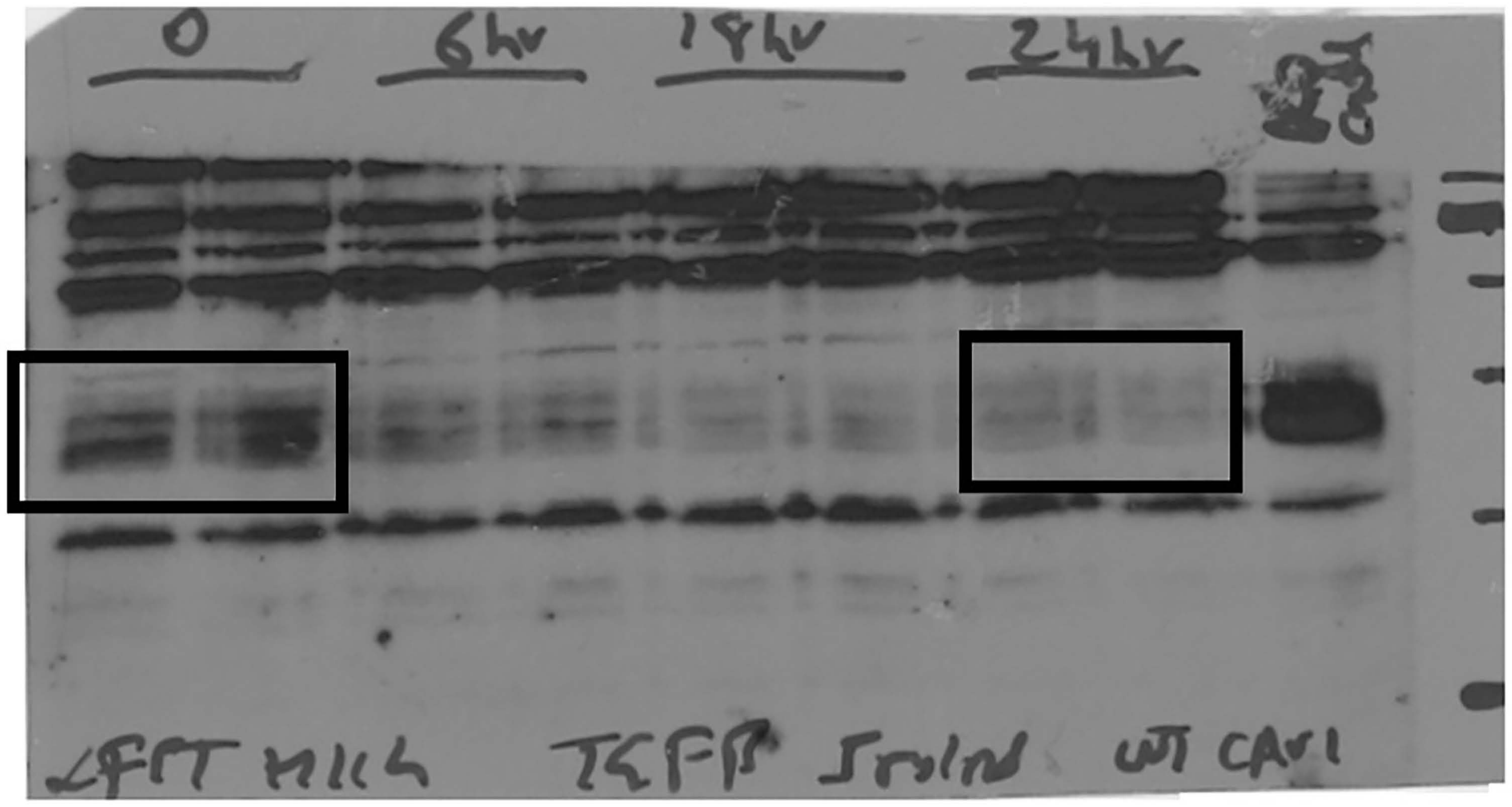

FST

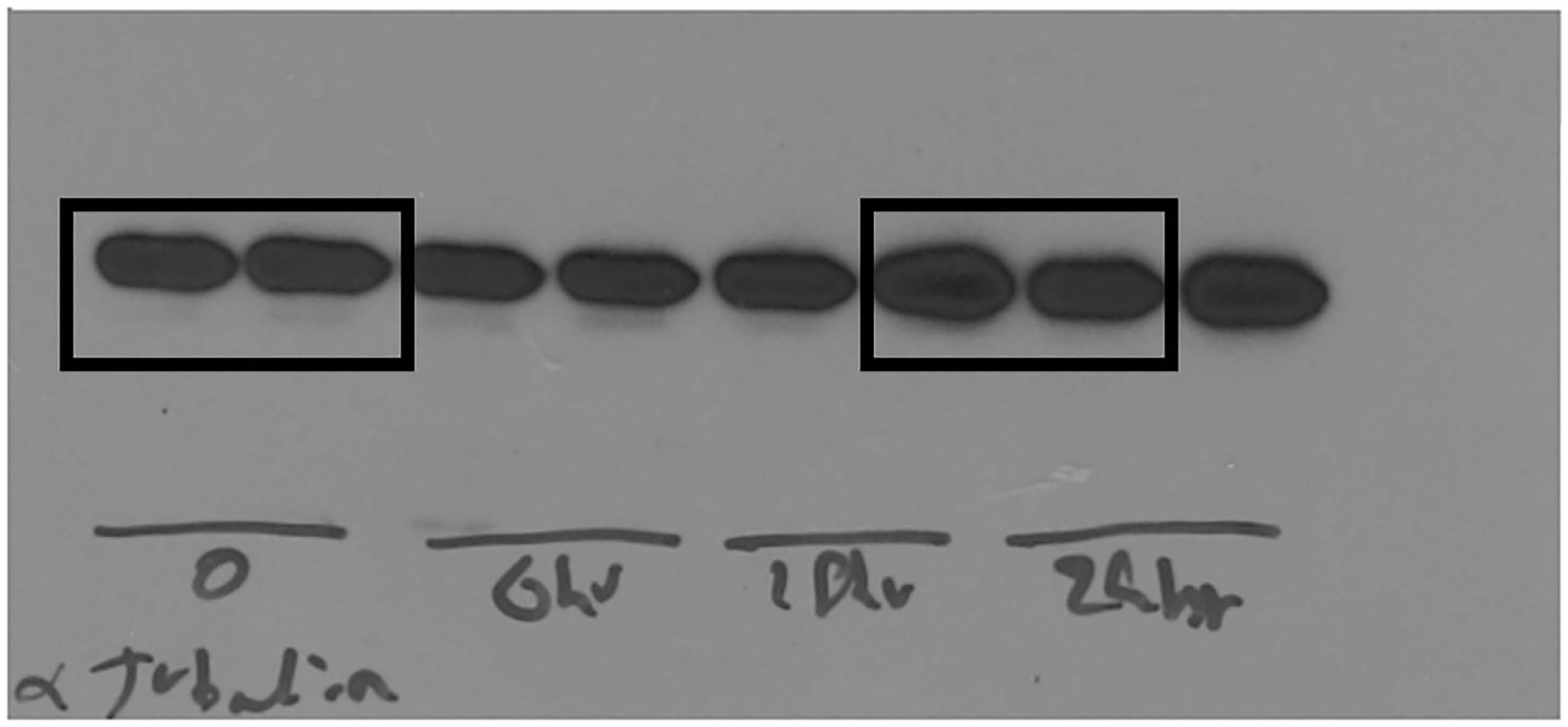

Tubulin

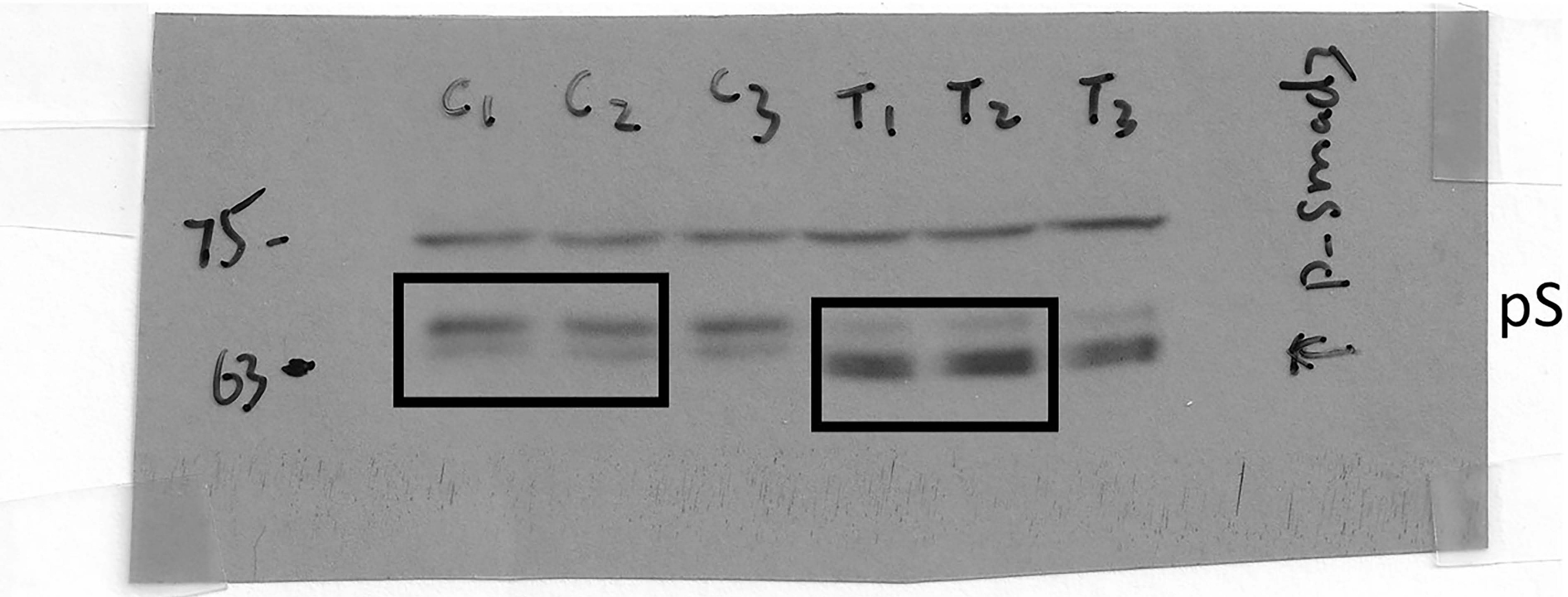

pSMAD3

3E

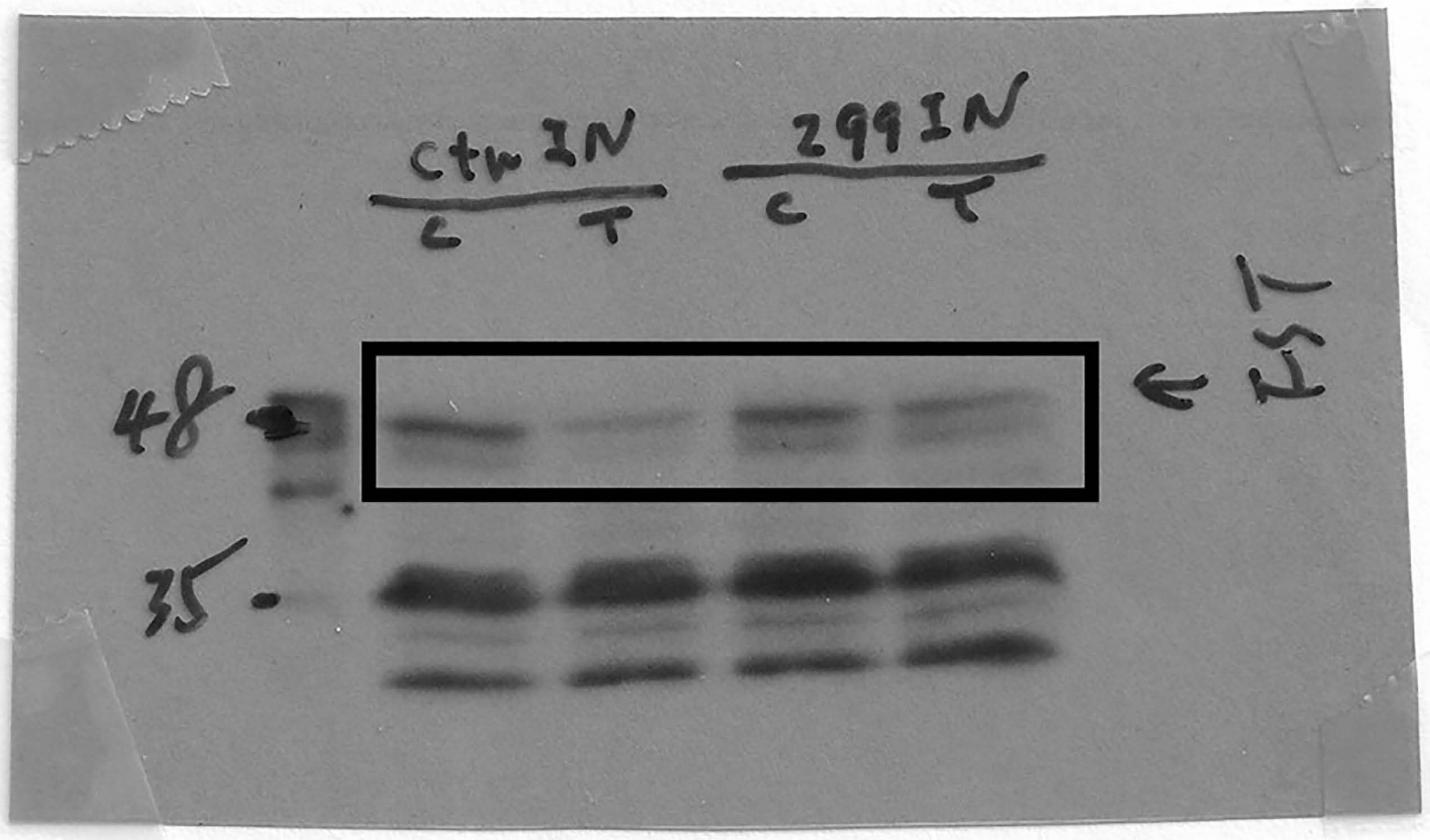

FST

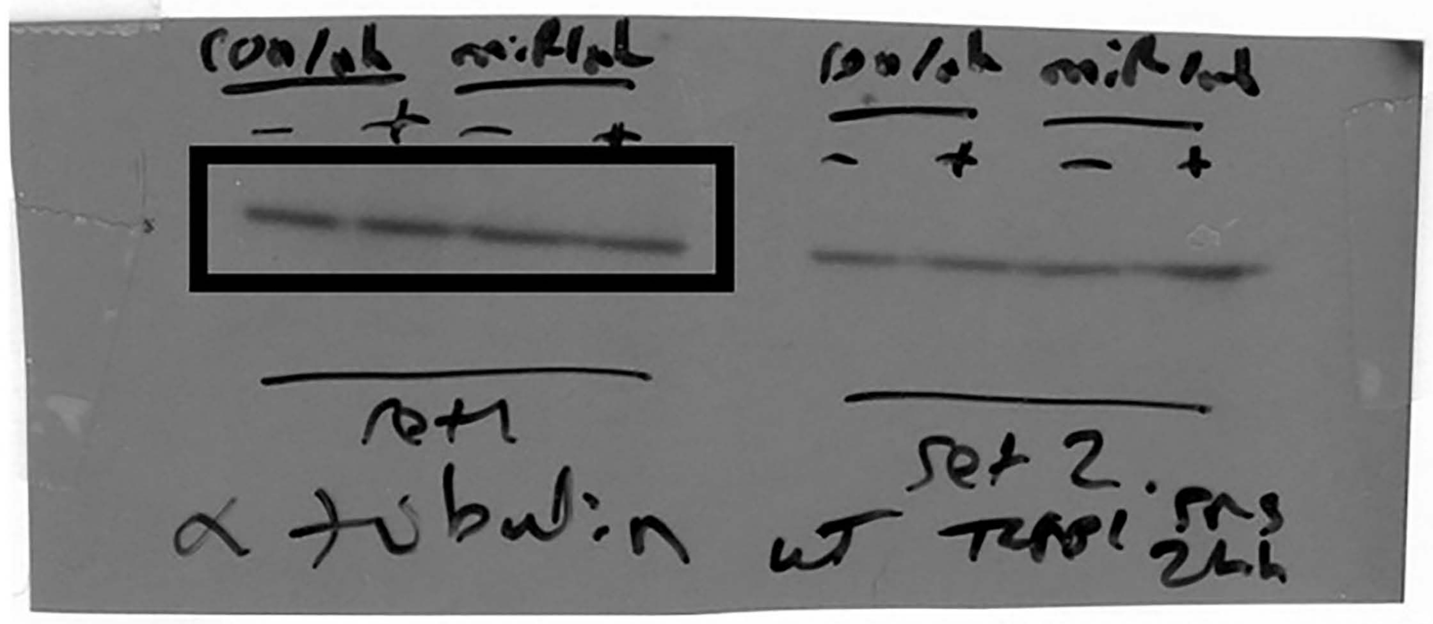

Tubulin

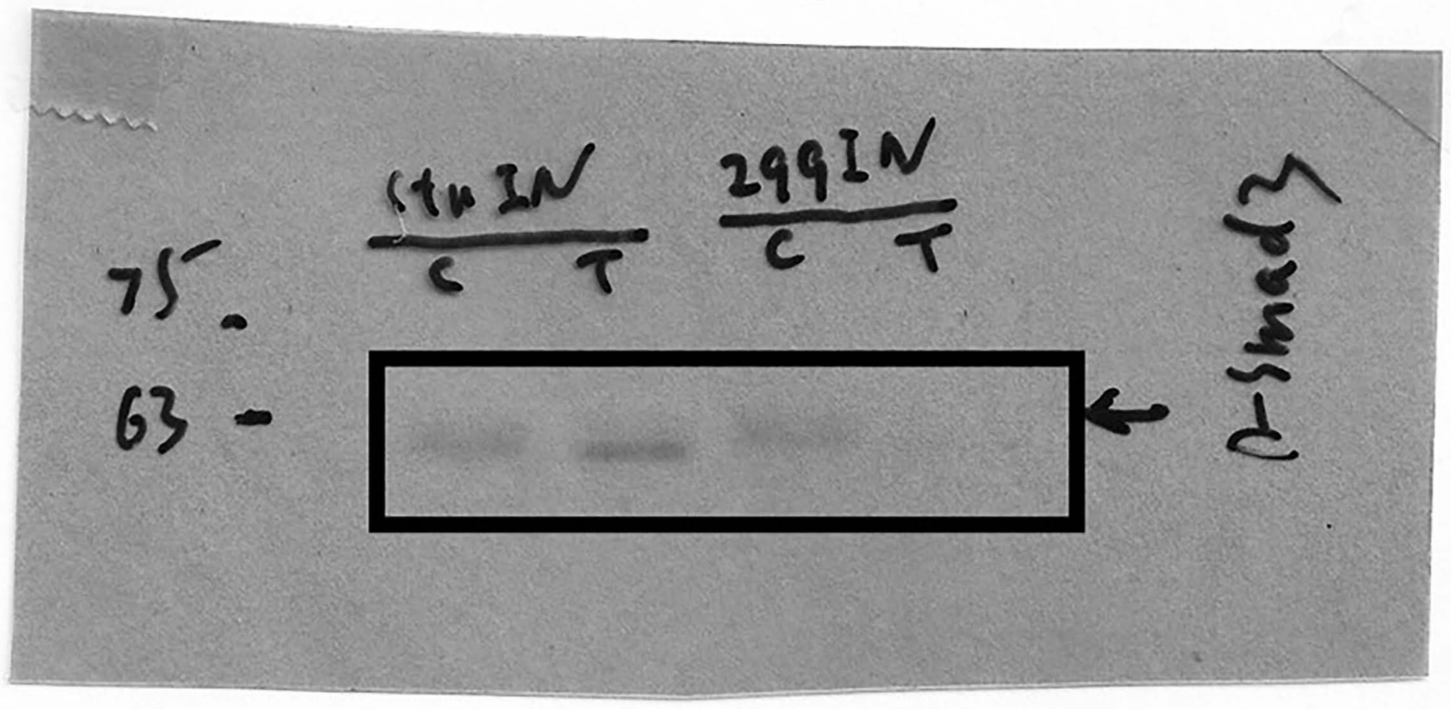

pSMAD3

Fig S9

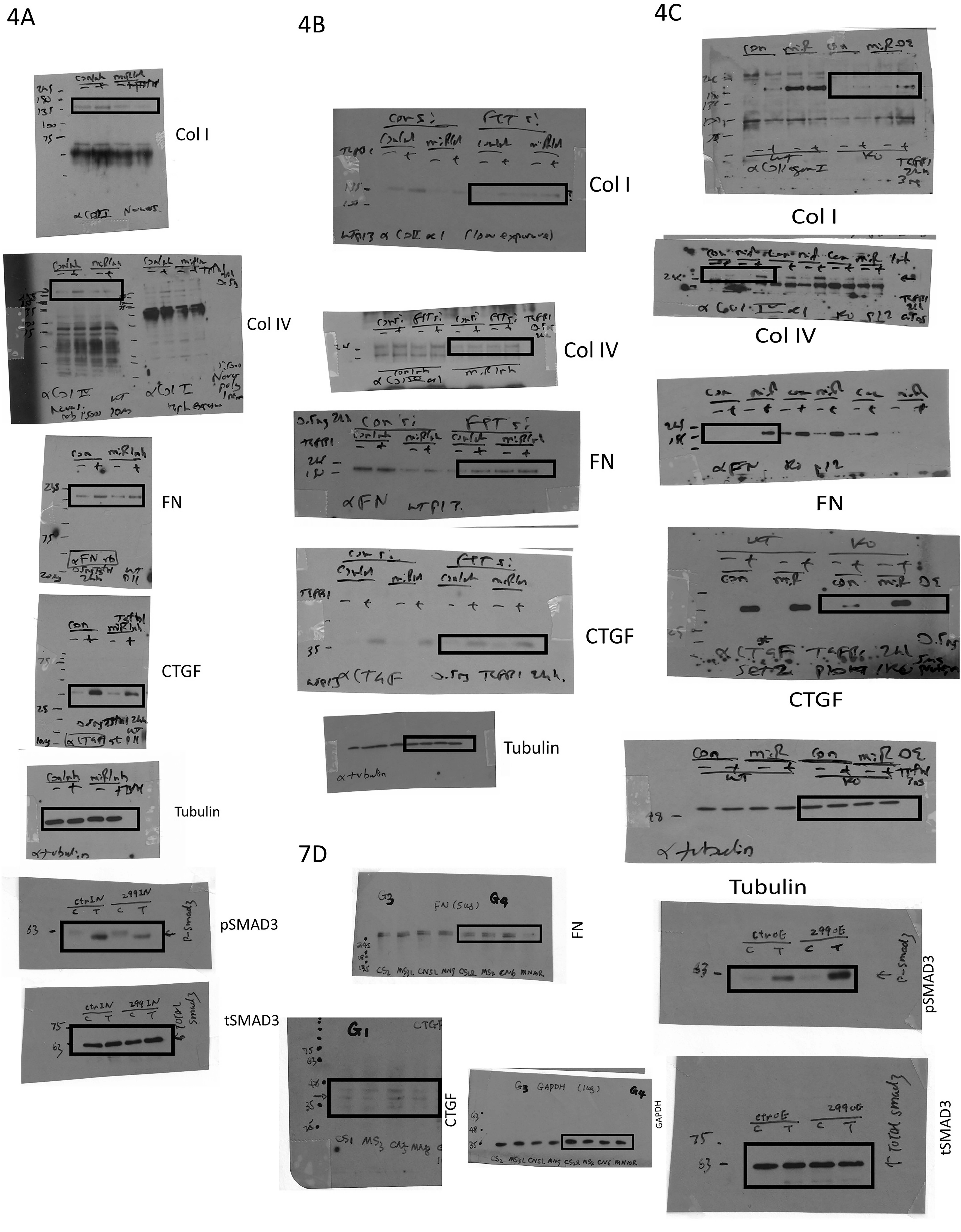
4A
Col I
Col IV
FN
CTGF
Tubulin
pSMAD3
tSMAD3
4B
Col I
Col IV
FN
CTGF
Tubulin
4C
Col I
Col IV
FN
CTGF
Tubulin
pSMAD3
tSMAD3
7D
FN
CTGF
GAPDH

Supplemental Tables

Table 1. Drugs and recombinant proteins.

Table 2. siRNA and miR LNA anti-miR probes for ISH and in vivo inhibition.

Table 3. Plasmids.

Table 4. Antibodies.

Table 5. qPCR Primers

Table 6. Synthesized oligonucleotide sequences for the cloning of mir299a-5p MRE-luc with ~60bp flanking the FST 3'UTR (highlighted in green) in PGL3 Control/ Vector (mir299a-5p 8mer MRE highlighted in yellow. (P=phosphate).

Table 1. Drugs and recombinant proteins.

Drugs and Recombinant Proteins	Dilution/Amount	Source
**Recombinant Mouse TGFß1**	0.5ng/ml-5ng/ml	R&D Systems
**Recombinant Mouse Follistatin 288**	0.1ug/ml - 1µg/ml	R&D Systems

Table 2. siRNA and miR LNA probes for ISH and in vivo inhibition.

siRNA	Application	Amount	Source
**Mouse Follistatin Silencer Select SiRNA**	Transfection	50nM	Life Tech
**Control Silencer Select SiRNA**	Transfection	50nM	Life Tech
**5'/3' DIG mmu-miR299a-5p miRCURY LNA detection probe**	ISH	2.5pmol	Exiqon/Qiagen; YD00615334
**5' DIG U6 miRCURY LNA detection probe, positive control**	ISH	2.5pmol	Exiqon/Qiagen; YD00699002
***In vivo* LNA anti-miR Inhibitor targeting mmu-miR299a-5p**	Mice treatment (SQ)	2mg/kg (2x/week post Nx, 1 dose pre Nx)	Exiqon/Qiagen; 339203
***In vivo* LNA anti-miR Inhibitor Scramble Control**	Mice treatment (SQ)	2mg/kg (2x/weekpost Nx, 1 dose pre Nx)	Exiqon/Qiagen; 339203

Table 3. Plasmids.

Construct	Source
**hFST 3´UTR Luciferase**	Dr. Zhengping Xu
**CAGA12 Luciferase**	Dr. Maree Bilandzic
**miR299a-5p precursor clone (GFP)**	Genecopoeia; MmiR3388-MR04
**miRNA scrambled precursor control clone (GFP)**	Genecopoeia; CmiR0001-MR04
**mir299a-5p MRE-Luc**	Generated in Lab
**miR299a-5p inhibitor clone (mCherry)**	Genecopoeia; MmiR-AN0369-AM02
**miRNA inhibitor scrambled control clone (mCherry)**	Genecopoeia; CmiR-AN0001-AM02

Table 4. Antibodies.

Antibody/Probe	Application	Dilution/Amount	Source
**Caveolin-1**	WB	1:1000	BD Biosci; 610059
**Follistatin (H-114)**	WB/IHC	1:1000/1:100	Santa Cruz; sc-30194
**Fibronectin**	WB/IHC	1:1000/1:200	Sigma; F3648
**Collagen Iα1**	WB	1:1000	Novus; NB600-408
**Collagen IVα1**	WB	1:1000	Novus; NB120-6586
**CTGF(G-14)**	WB	1:1000	Santa Cruz; sc-34772
**GFP (4B10)**	WB	1:1000	Cell Signaling; 2955
**α-Tubulin**	WB	1:10,000	Sigma; T6074
**GAPDH**	WB	1:10,000	CST; 2118
**Nephrin**	IF	1:10,000	R&D; AF3159
**CD31**	IHC	1:200	Cell Signaling; 77699
**F4/80**	IHC	1:500	Biorad; MCA497R
**CD3**	IHC	1:300	Dako; A0452
**pSMAD3**	IHC, WB	1:3000, 1:2000	Novus; NBP1-77836
**tSMAD3**	WB	1:2000	Abcam; ab-40854
**α8-integrin**	IF	1:200	R&D; BAF4076
**Ki67**	IHC	1:5000	Abcam; ab15580
**anti-Digoxigenin-AP Fab fragment**	ISH	1:500	Roche; 11093274910

Table 5. qPCR Primers.

Primer	Forward	Reverse
**mmu-miR-299a-5p**	TGGTTTACCGTCCCACATACAT	Universal – Proprietary (Quanta)
**mmu-miR-380-5p**	ATGGTTGACCATAGAACATGCG	Universal – Proprietary (Quanta)
**mmu-miR-384-5p**	TGTAAACAATTCCTAGGCAATGT	Universal – Proprietary (Quanta)
**mmu-miR-425-5p**	AATGACACGATCACTCCCGTTGA	Universal – Proprietary (Quanta)
**mmu-miR-489-3p**	AATGACACCACATATATGGCAGC	Universal – Proprietary (Quanta)
**mmu-miR-568**	ATGTATAAATGTATACACAC	Universal – Proprietary (Quanta)
**mmu-miR-504-3p**	AGGGAGAGCAGGGCAGGGTTTC	Universal – Proprietary (Quanta)
**mmu-miR-505-3p**	CGTCAACACTTGCTGGTTTTCT	Universal – Proprietary (Quanta)
**mmu-miR-6384**	GCTTTCCTACTGTTTCCCTG	Universal – Proprietary (Quanta)
**mmu-miR-690**	AAAGGCTAGGCTCACAACCAAA	Universal – Proprietary (Quanta)
**mmu-miR-7226-3p**	TGACACAGCCATTCTCTGAGCAG	Universal – Proprietary (Quanta)
**mmu-miR-7232-3p**	TGGTTGAATTCGACTTTGGGGC	Universal – Proprietary (Quanta)
**mmu-miR-878-3p**	GCATGACACCACACTGGGTAGA	Universal – Proprietary (Quanta)
**mmu-miR-882**	AGGAGAGAGTTAGCGCATTAGT	Universal – Proprietary (Quanta)
**Mouse U6 snRNA**	TGGCCCCTGCGCAAGGATG	Universal – Proprietary (Quanta)
**Mouse FST**	AAAACCTACCGCAACGAATG	GGTCTGATCCACCACACAAG
**Mouse 18S rRNA**	GCCGCTAGAGGTGAAATTCTTG	CATTCTTGGCAAATGCTTTCG

Table 6. Synthesized oligonucleotide sequences for the cloning of mir299a-5p MRE-luc with ~60bp flanking the FST 3'UTR (highlighted in green) in PGL3 Control/ Vector (mir299a-5p 8mer MRE highlighted in yellow. (P=phosphate).

miR299a-5p MRE Luciferase

Forward Strand/Sense	Reverse Strand/Anti-Sense
*P*ctagagttttttttttccccctttgtaaaccatttaagtccattcctcact atgcacacccacctgtt	*P*ctagaacaggtgggtgtgcatagtgaggaat ggacttaaatggtttacaagggggaaaaaaaaaact
